# Distinct Co-methylation Patterns in African and European Populations and Their Genetic Associations

**DOI:** 10.1093/gpbjnl/qzaf096

**Published:** 2025-03-22

**Authors:** Zheng Dong, Nicole Gladish, Maggie P Y Fu, Samantha L Schaffner, Keegan Korthauer, Michael S Kobor

**Affiliations:** Centre for Molecular Medicine and Therapeutics, BC Children’s Hospital Research Institute, University of British Columbia, Vancouver, BC V5Z 4H4, Canada; Genome Science and Technology Graduate Program, University of British Columbia, Vancouver, BC V5Z 4S6, Canada; Centre for Molecular Medicine and Therapeutics, BC Children’s Hospital Research Institute, University of British Columbia, Vancouver, BC V5Z 4H4, Canada; Department of Medical Genetics, University of British Columbia, Vancouver, BC V5Z 4H4, Canada; Centre for Molecular Medicine and Therapeutics, BC Children’s Hospital Research Institute, University of British Columbia, Vancouver, BC V5Z 4H4, Canada; Genome Science and Technology Graduate Program, University of British Columbia, Vancouver, BC V5Z 4S6, Canada; Centre for Molecular Medicine and Therapeutics, BC Children’s Hospital Research Institute, University of British Columbia, Vancouver, BC V5Z 4H4, Canada; Department of Medical Genetics, University of British Columbia, Vancouver, BC V5Z 4H4, Canada; Centre for Molecular Medicine and Therapeutics, BC Children’s Hospital Research Institute, University of British Columbia, Vancouver, BC V5Z 4H4, Canada; Department of Statistics, University of British Columbia, Vancouver, BC V6T 1Z4, Canada; Centre for Molecular Medicine and Therapeutics, BC Children’s Hospital Research Institute, University of British Columbia, Vancouver, BC V5Z 4H4, Canada; Department of Medical Genetics, University of British Columbia, Vancouver, BC V5Z 4H4, Canada; Edwin S.H. Leong Centre for Healthy Aging, University of British Columbia, Vancouver, BC V6T 1Z3, Canada

**Keywords:** Epigenetic variation, Ancestry, Whole-genome bisulfite sequencing, Immune, Metabolism

## Abstract

Human populations have substantial genetic diversity, but the extent of epigenetic diversity remains unclear, as population-specific DNA methylation (DNAm) has only been studied for ∼ 3.0% of CpGs. In this study, we quantified DNAm using whole-genome bisulfite sequencing (WGBS) and analyzed it alongside whole-genome genotype data to provide a more comprehensive view of population-specific DNAm. Using a co-methylated region (CMR) approach, 36,657 CMRs were identified in WGBS data from 62 lymphoblastoid B-cell line (LCL) samples, with subsequent validation in a combined array dataset of 326 LCL samples. Between individuals of European and African ancestry, 101 CMRs exhibited population-specific DNAm patterns (Pop-CMRs), including 91 Pop-CMRs not reported in previous investigations. These regions spanned genes (*e.g.*, *CCDC42*, *GYPE*, *MAP3K20*, and *OBI1*) related to diseases (*e.g.*, malaria infection and diabetes) with differing prevalence and incidence between populations. Over half of the Pop-CMRs were associated with genetic variants, displaying population-specific allele frequencies and primarily mapped to genes involved in metabolic and infectious processes. Additionally, subsets of Pop-CMRs were applicable in East Asian populations and peripheral blood-based tissues. This study highlights genome-wide DNAm differences between populations and examines their associations with genetic varation and biological relevance, advancing our understanding of epigenetic contributions to population specificity.

## Graphical abstract

**Figure:**
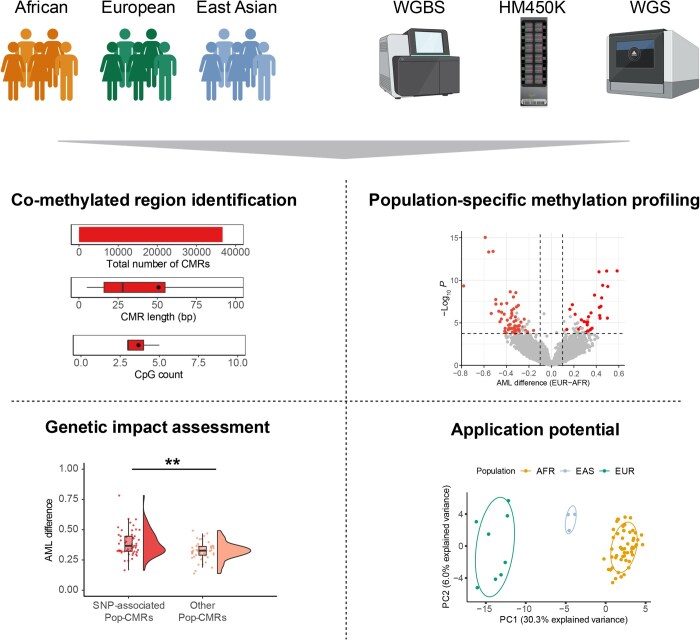

## Introduction

Human populations defined on the basis of ancestry exhibit extensive phenotypic and molecular diversity as a result of environmental adaptations, including differences in immune function and disease susceptibility [1,2]. In the context of evolutionary biology, genetic variation plays an important role in the adaptation of individual human populations and contributes to phenotypic differences between populations [2–5]. Beyond genetic variation, epigenetic modifications may also contribute to diversity in key biological processes not fully explained by genetic variation, as epigenetic variation has been shown to influence development, disease, and responses to various environmental conditions [6–8]. DNA methylation (DNAm) is a highly stable and widespread epigenetic mark that has been extensively studied in relation to inter-population epigenetic differences and the population-specific DNAm landscape [9–17]. For example, in lymphoblastoid B-cell line (LCL) samples from 5 diverse human populations, population-specific DNAm measured at 485,000 CpGs mirrors genetic diversity and can more strongly reflect genetic influence than transcriptomic-level measurements [11].

However, these previous studies can be improved with more advanced technologies and analytical approaches. First, all were conducted using various iterations of DNAm arrays that cover only a small fraction of the approximately 28,000,000 CpG sites in the human genome. For example, the majority of earlier epigenome-wide association studies (EWASs) investigating human population specificity used the HM450K array, which covers ∼ 1.7% of genomic CpGs. This sparse coverage hinders assessment of genome-wide DNAm differences between populations and exploration of the genetic impact of DNAm diversity. In contrast, whole-genome bisulfite sequencing (WGBS) is a powerful method of assessing DNAm patterns across the entire human genome, covering up to ∼ 95% of all CpG sites. This increased coverage offers a more comprehensive representation of the methylation state and captures finer details of population-specific variation. Second, previous studies focused on the population specificity of individual CpG sites. However, the deposition, maintenance, and removal of DNAm are predominantly controlled by DNA methyltransferases and ten-eleven translocation (TET) methylcytosine dioxygenases, which catalyze methylation changes regionally rather than at the single-site level, forming regions of CpGs with correlated methylation status [18,19]. As genomic regions containing regulatory elements, such as gene promoters and CpG islands, are characterized by high CpG density, region-based analyses are likely to have greater functional utility than single-CpG-based studies [20,21]. Region-based approaches can also reduce the multiple testing burden, by as much as 30-fold through data reduction, combining many adjacent CpGs into a single unit [21]. Our group previously developed a method called Co-Methylation with genomic CpG Background (CoMeBack), which performs genome-wide identification of co-methylated regions (CMRs), defined as regions of adjacent CpG sites with highly correlated DNAm status across individuals [21]. In contrast to differentially methylated regions (DMRs), which are identified using phenotypic information, CMR identification relies solely on the correlation structure of adjacent CpG sites and does not incorportate any phenotypic data [21,22]. Therefore, using a region-based approach to evaluate genome-wide DNAm differences measured by WGBS between human populations could improve the discovery of population-specific DNAm alterations and provide insight into biological processes underlying population diversity.

In this study, we quantified DNAm using WGBS data and analyzed it using the CMR approach to obtain a comprehensive view of population-specific DNAm. We identified unique population-specific DNAm patterns that were not captured in previous array-based studies and observed relationships among population-specific DNAm, genetic variation, and potential biological relevance. This research broadens our understanding of the crucial roles that epigenetic variation plays in population specificity.

## Results

### CMRs were extensive, reliable, and enriched in transcription regulatory elements

**Figure 1:**
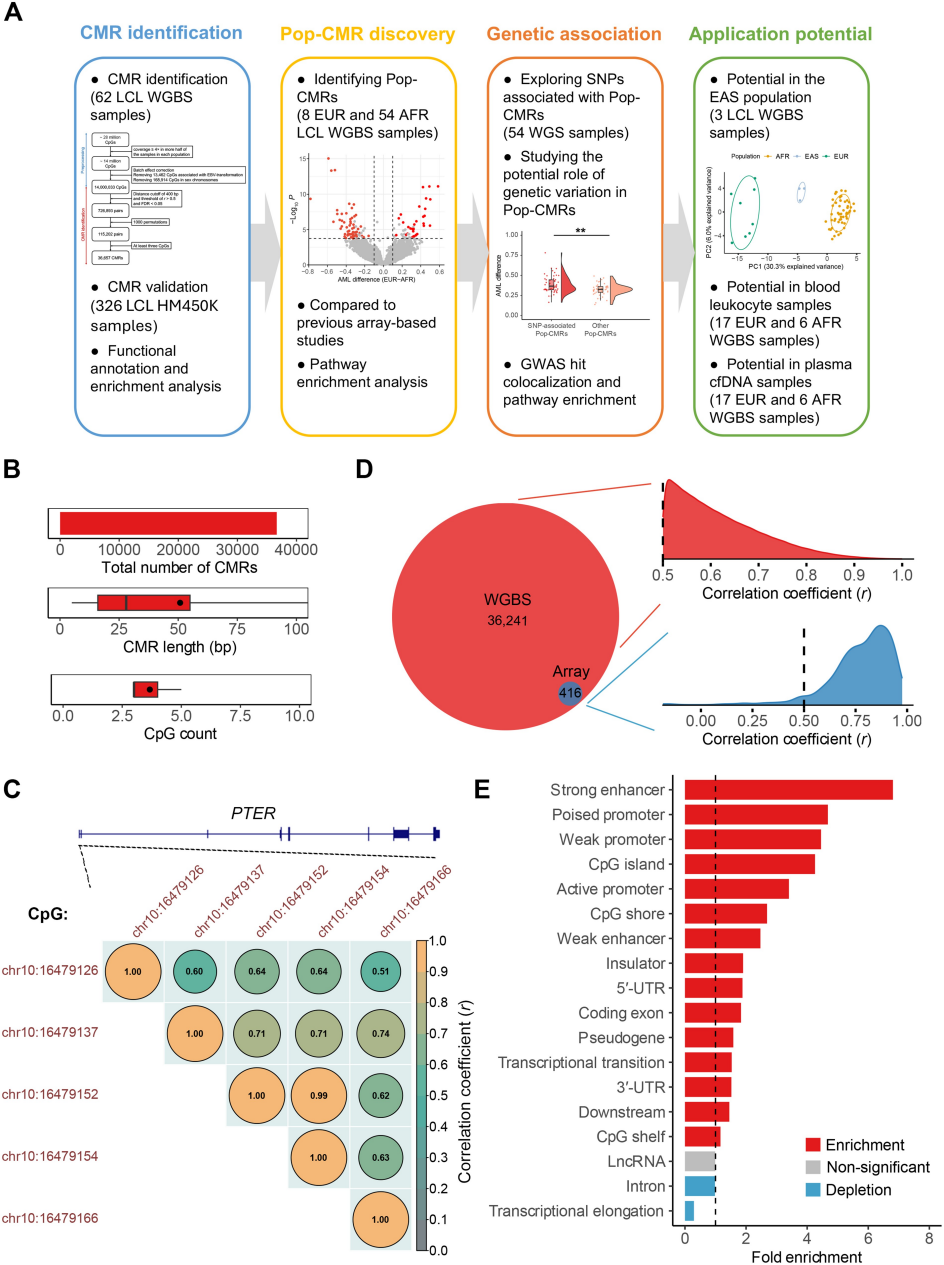
Identification and characterization of CMRs in WGBS samples **A**. Flowchart of CMR identification and population specificity analysis in multi-omics data. **B**. CMR characteristics evaluated by total number of CMRs identified, distribution of CMR length, and distribution of CpG counts in the CMRs (top to bottom). **C**. An example of a CMR containing five CpG sites located within the promoter of *PTER*. The correlation coefficiets (*r*) between adjacent CpG sites are colored-coded. CpGs are included in a CMR if they are proximal and have *r* > 0.5 and FDR < 0.05. **D**. Density plots of correlation coefficients amongst CpGs within a given CMR identified in the WGBS dataset (EUR *n* = 8, AFR *n* = 54; top) and in a combined HM450K dataset comprised of 326 LCL samples (EUR *n* = 157, AFR *n* = 169; bottom). Venn diagram shows CMRs identified by WGBS and those validated by the HM450K array. **E**. Significant enrichment (red) or depletion (blue) of CMRs in genomic elements (FDR < 0.05). A dashed vertical line indicates the fold enrichment of 1. Fold enrichment was calculated based on random sampling repeated 1,000,000 times. For each set of CMRs enriched in a certain genomic element, the regions with matched CpG count and density were randomly selected from genomic sequences with a minimum mapping depth of 4 and without EBV transformation-associated and sex chromosome CpGs. CMR, co-methylated region; AFR, African; EAS, East Asian; EUR, European; cfDNA, cell-free DNA; GWAS, genome-wide association study; LCL, lymphoblastoid B-cell line; Pop-CMR, population-specific CMR; WGS, whole-genome sequencing; WGBS, whole-genome bisulfite sequencing; FDR, false discovery rate; *PTER*, phosphotriesterase-related; EBV, Epstein–Barr virus.

WGBS data from 62 LCL samples, drived from 54 individuals of African (AFR) ancestry and 8 individuals of European (EUR) ancestry, were analyzed to identify CMRs (**Figure 1**A; data sources are described in Table S1) [23–28]. DNAm patterns of CpGs from sequencing reads were extracted from all samples and filtered using a 4× coverage threshold (Figure S1). CpGs with DNAm changes associated with Epstein–Barr virus (EBV) immortalization of the LCLs were excluded based on prior findings [28]. Next, the local correlation structure of adjacent CpG methylation (*r*) was calculated using Pearson correlation across all samples. CMRs were defined as genomic regions containing at least three highly correlated CpG sites with inter-CpG distances < 400 bp, a correlation threshold of *r* > 0.5, and false discovery rate (FDR) < 0.05. These criteria were stricter than previous studies, which defined CMRs as regions with at least two correlated CpGs within 400 bp and *r* > 0.3, without applying an FDR threshold [21,29]. To minimize the impact of unequal sample sizes, correlation structure within each CMR was further validated by selecting a random subset of AFR samples equal in number to EUR samples, applying the same Pearson’s *r* and Benjamini–Hochberg FDR thresholds, and repeating this process 1000 times (see the “Identification of CMRs from LCL WGBS” section in Materials and methods).

A total of 36,657 CMRs, overlapping with 9859 genes, were identified across the 62 WGBS samples with an average block size of 50.81 bp and a mean CpG count of 3.67 (Figure 1B, Figure S2; CMRs are listed in Table S2). By design, DNAm levels of CpGs within each CMR are highly correlated, illustrated by an example CMR (chr10:16479126–16479166) containing five adjacent CpGs in the promoter of the phosphotriesterase-related (*PTER*) gene with correlation coefficients (*r*) ranging from 0.51 to 0.99 (Figure 1C). Notably, the majority of identified CMRs (99.1%) were not covered by CpG probes on the HM450K array, the platform most commonly used in studies characterizing population-specific DNAm, underscoring the added value of our study in advancing the understanding of population-specific DNAm.

How well CMRs can be reproduced using DNAm profiling techniques and DNA sources not included in the dataset used for CMR identification remains to be determined. To address this, we further validated these CMR correlation structures using a combined HM450K dataset consisting of 326 LCL samples from three individual datasets from the Gene Expression Omnibus (GEO: GSE36369, GSE39672, and GSE40699; EUR *n* = 157, AFR *n* = 169) (Table S3) [12,14,30]. Using the same thresholds (*r* > 0.5 and FDR < 0.05), 94.8% of the 416 CMRs that could be evaluated on the array (those with at least two CpGs, given the array’s sparse coverage; total CpGs *n* = 1033) showed significant correlation. This supports the reliability of the CMRs identified in the WGBS data (Figure 1D).

Functional characterization of the genomic features encompassed within the identified CMRs was conducted by mapping them to 18 canonical genomic elements retrieved from the UCSC Genome Browser and GENCODE database [31]. The majority of CMRs (*n* = 29,792; 81.27%) were found to overlap with these genomic elements, particularly introns, strong enhancers, CpG shores, and weak enhancers (Table S4). To address the enrichment of CMRs in specific genomic elements, we compared them to 1,000,000 sets of randomly selected regions matched for CpG count and density (by maintaining the maximum distance of < 400 bp between adjacent CpGs, as used in CMR identification). This analysis revealed significant enrichment of CMRs in 15 genomic features, particularly in canonical regulatory elements. Notably, we observed more than 5-fold enrichment of CMRs in strong enhancers, while CMRs were significantly depleted in transcriptional elongation regions and introns (all FDR < 0.05) (Figure 1E; Table S4). The enrichment of CMRs in canonical regulatory elements such as promoters and enhancers aligns with findings from a previous study using whole-blood HM450K array data [21]. This consistency highlights a major strength of the CMR approach: it may more accurately reflect functionally relevant methylation patterns than analyses based on individual CpG sites. Thus, by employing this approach, our study likely captures local DNAm patterns that are biologically meaningful and either directly or indirectly involved in transcriptional regulation.

### Comparable global CMR methylation profiles between EUR and AFR populations

To compare global DNAm patterns between human populations, we analyzed genome-wide CMR methylation differences between EUR and AFR populations. For each CMR, DNAm levels were calculated as the average methylation level (AML) of correlated CpG sites within the region. Globally, the two populations (EUR *n* = 8, AFR *n* = 54) exhibited highly similar DNAm patterns (cosine similarity = 0.98; average absolute DNAm difference = 8.87 × 10^−3^; Mann–Whitney U test *P* = 0.47). Principal component analysis (PCA) of CMR DNAm also showed no global seperation between populations (Figure S3). Moreover, no significant DNAm differences were observed across genomic features between populations (cosine similarity ranged from 0.96 to 0.99; average absolute DNAm difference ranged from 1.60 × 10^−4^ to 0.028, depending on genomic feature; all Mann–Whitney U test FDR > 0.05) (Table S5). These findings demonstrate a high degree of similarity in global CMR methylation patterns between individuals of EUR and AFR ancestry and suggest minimal influence of technical artifacts, such as batch effects or sample processing differences, on the observed results.

### Identification of population-specific CMRs with a trend toward higher methylation in AFR compared to EUR

The main goal of this study was to test the hypothesis that subtle, yet important CMR DNAm differences exist between EUR and AFR populations. To this end, linear regression adjusting for sex was used to identify population-specific CMRs (Pop-CMRs) (see the “Detection and validation of Pop-CMRs” section in Materials and methods). We further tested whether there were any systematic biases in our results by estimating the inflation factor (λ) using the BACON R package, a tool established for estimation and correction of bias and inflation in EWASs and transcriptome-wide association studies (TWASs) [32]. We observed an inflation factor of 1.08, suggesting that systemic bias is unlikely. A strict absolute AML difference threshold of 0.1 between EUR and AFR populations, together with an FDR cutoff of 0.05, was applied to minimize the false positive rate [33]. Potential effects of unbalanced sample sizes and subpopulation structure were assessed using 1000 bootstrap replicates and 1000 permutation tests, respectively. Thirty-five CMRs that passed the AML difference and FDR thresholds were subsequently removed due to the possible effects of unbalanced sample sizes (*n* = 34) and subpopulations (*n* = 1).

**Figure 2:**
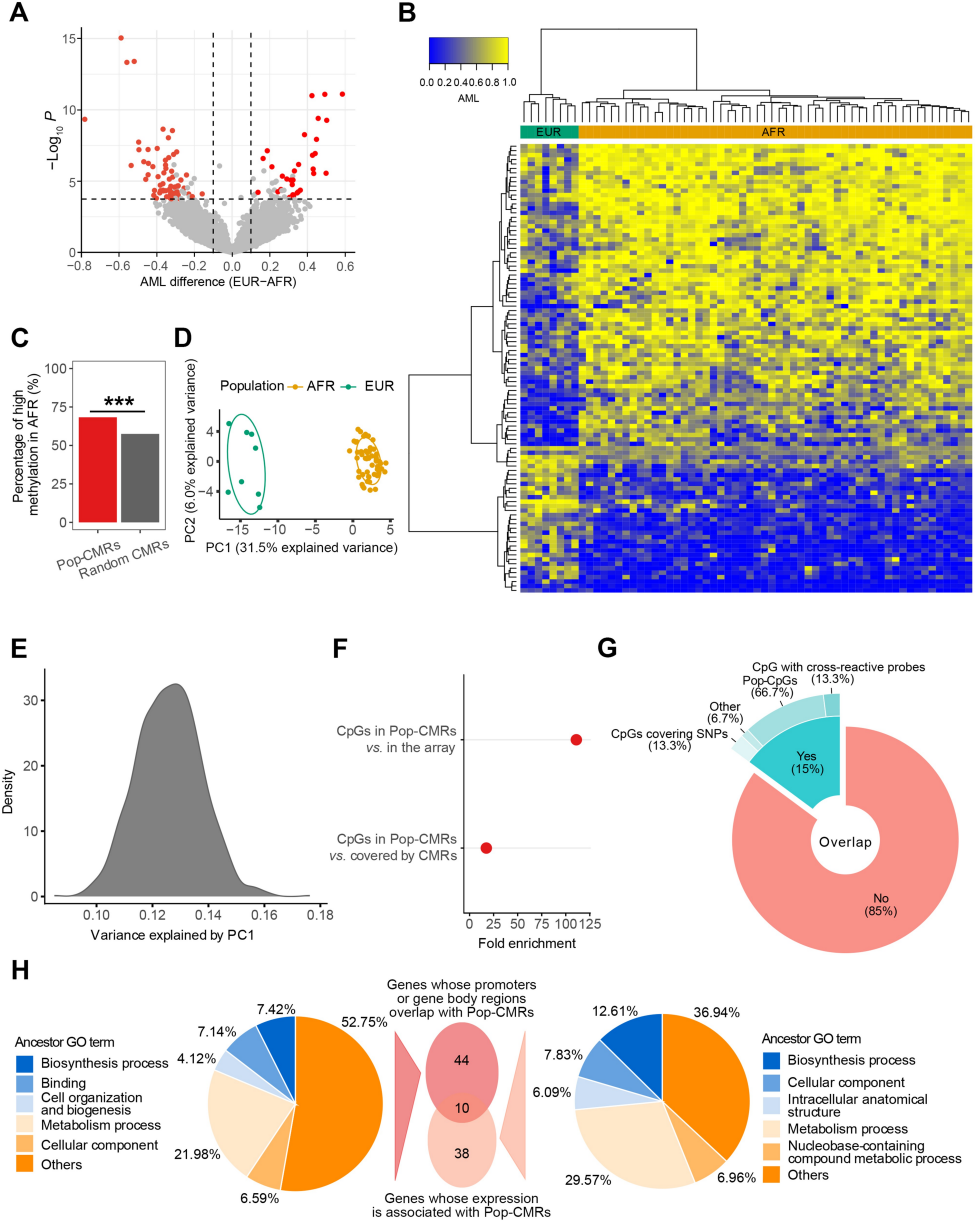
Identification and characterization of Pop-CMRs between EUR and AFR individuals Data from all 62 LCL WGBS samples (EUR *n* = 8, AFR *n* = 54) used in this study are shown. **A**. Volcano plot of significant Pop-CMRs. The plot shows AML difference (x-axis) and significance (y-axis) between EUR and AFR individuals, adjusted for sex. Significant Pop-CMRs are shown in red; CMRs that were non-significant or affected by an unbalanced sample size or subpopulation structure are shown in gray. Each spot represents one CMR. **B**. Unsupervised hierarchical clustering heatmap of DNAm patterns at 101 Pop-CMRs in EUR and AFR LCL samples. **C**. Percentage of more highly methylated Pop-CMRs in AFR *vs*. EUR samples compared to random CMR sets. ***, *P* < 0.001 (1000 bootstrap replicates). **D**. Loadings on the first two PCs for the 101 Pop-CMRs for each LCL WGBS sample, color-coded by population. **E**. Density plot showing the proportion of variance explained by PC1 from AMLs of randomly selected CMRs. Random sampling was repeated 1000 times. Only the explained variances for the random CMR sets are shown, as these were used to compare the variance explained by PC1 of Pop-CMRs. **F**. Enrichment of array CpGs covered by Pop-CMRs showing significant DNAm differences between EUR and AFR populations, compared to CpGs randomly selected from all CpGs in the array or CpGs in the array covered by CMRs. Random sampling was repeated 1000 times for each comparison. Both *P* < 0.001 (1000 bootstrap replicates). **G**. Pie-donut chart showing the overlap of 101 Pop-CMRs with array CpGs from HM27K, HM450K, and EPIC. Most of the overlapping CpGs were previously identified as Pop-CpGs. **H**. GO classification. Left: ancestor GO terms from GO enrichment analysis of 54 genes whose promoters or gene bodies overlap with Pop-CMRs. Right: ancestor GO terms from GO enrichment analysis of 48 genes whose expression is associated with Pop-CMRs in the iMETHYL database. The overlap between these two gene sets is illustrated by the central Venn diagram. Detailed pathway information is provided in Tables S14 and S16. DNAm, DNA methylation; PC, principal component; var, variance; AML, average methylation level; GO, Gene Ontology.

Using this approach, 101 Pop-CMRs were identified, showing significant DNAm differences between the two populations (**Figure 2**A and B; **Table 1**). Of them, 69 (68.3%) exhibited higher methylation in AFR compared to EUR samples (*i.e.*, DNAm levels were higher in AFR by any magnitude) (Figure 2C). This proportion was significantly greater than 57.5% observed by random sampling among all CMRs in AFR samples (1000 bootstrap replicates, *P* < 0.001) (Figure 2C). This trend was consistent with findings from a previous array-based study, albeit the effect size was smaller in the current study (10.8% in our study *vs*. 22.3% in the previous study) [9], potentially due to differences in cell type (LCL *vs*. monocyte) and/or DNAm profiling platforms (WGBS *vs*. EPIC). We next performed PCA on the 101 Pop-CMRs to assess DNAm variation across samples. The two populations were clearly distinct along the first principal component (PC1) loadings, accounting for 31.5% of the total variance (Figure 2D). As expected, PC1 for Pop-CMRs outperformed random CMR sets in distinguishing between populations, explaining 2.5-fold more variance on average (31.5% *vs*. 12.5%; 1000 bootstrap replicates, *P* < 0.001) (Figure 2E). Annotation of the Pop-CMRs using the EWAS Catalog identified CpG sites previously associated with population-specific traits and diseases, including pancreatic ductal adenocarcinoma, type 2 diabetes, and breastfeeding [34]. Notably, breastfeeding-associated CpGs were significantly enriched within Pop-CMRs relative to random CMRs (333.3-fold; FDR = 0.048) (Table S6). These findings suggest that the identified Pop-CMRs are likely reflect biologically meaningful population-specific differences rather than random variation.

**Table 1:** Methylation differences of Pop-CMRs across three human populations

Pop-CMR	AML			EUR *vs.* AFR		EUR *vs.* EAS		AFR *vs.* EAS		Previously reported population-specific CpG	Gene^*^
	EUR (*n* **=** 8)	AFR (*n* **=** 54)	EAS (*n* **=** 3)	AML difference	FDR	AML difference	FDR	AML difference	FDR		
chr1:105416732–105416911	0.47	0.14	0.45	0.33	3.99E−02	0.02	8.52E−01	−0.31	9.18E−02		
chr1:1329689–1329749	0.96	0.32	0.48	0.64	6.02E−08	0.49	3.45E−01	−0.16	3.38E−01		*CCNL2*
chr1:142801785–142801923	0.38	0.52	0.41	−0.14	2.85E−02	−0.03	7.60E−01	0.11	3.90E−01		
chr1:169131171–169131228	0.34	0.67	0.42	−0.34	2.02E−02	−0.09	6.43E−01	0.25	7.97E−02		*NME7*
chr1:205141231–205141245	0.56	0.85	0.78	−0.29	1.80E−02	−0.22	4.17E−01	0.07	3.54E−01		*DSTYK*
chr1:205819126–205819423	0.62	0.12	0.26	0.50	6.02E−08	0.36	3.45E−01	−0.14	2.74E−01	cg11965913, cg14159672, cg14893161, cg05841700, cg07533224, cg12898220	*PM20D1*
chr1:206288248–206288273	0.22	0.06	0.22	0.16	2.37E−02	0.00	9.20E−01	−0.16	7.97E−02		
chr1:218775950–218775974	0.40	0.73	0.63	−0.33	3.21E−02	−0.23	3.73E−01	0.10	5.01E−01		
chr1:36724301–36724326	0.55	0.84	0.63	−0.29	2.26E−02	−0.08	1.00E+00	0.21	1.42E−01		*THRAP3*
chr1:570123–570221	0.66	0.15	0.61	0.51	2.22E−06	0.05	1.00E+00	−0.46	4.95E−02		
chr1:58524114–58524138	0.16	0.61	0.30	−0.45	5.96E−04	−0.15	3.45E−01	0.31	5.84E−02		*DAB1*
chr1:71013831–71013852	0.18	0.54	0.51	−0.36	2.04E−02	−0.33	3.45E−01	0.03	8.53E−01		
chr10:118641228–118641250	0.64	0.88	0.73	−0.24	1.56E−02	−0.09	9.20E−01	0.15	7.97E−02		
chr10:128918479–128918639	0.25	0.55	0.34	−0.30	2.32E−02	−0.09	6.86E−01	0.21	1.70E−01		*DOCK1*
chr10:28791206–28791232	0.36	0.76	0.43	−0.40	1.04E−03	−0.07	7.60E−01	0.33	7.78E−02		
chr10:54504049–54504586	0.12	0.62	0.25	−0.50	4.52E−05	−0.13	7.60E−01	0.37	9.24E−02		
chr11:33910910–33910955	0.43	0.80	0.57	−0.37	8.89E−04	−0.14	4.17E−01	0.23	1.70E−01		*LMO2*
chr11:45840812–45841145	0.60	0.90	0.73	−0.30	9.59E−06	−0.13	6.86E−01	0.17	1.59E−01		
chr11:66362800–66362849	0.61	0.90	0.69	−0.29	3.84E−03	−0.08	6.86E−01	0.21	1.39E−01		*CCS*
chr11:82169987–82170030	0.12	0.56	0.24	−0.45	1.29E−04	−0.13	6.86E−01	0.32	1.49E−01		
chr11:9419206–9419271	0.38	0.67	0.51	−0.29	3.39E−02	−0.14	7.60E−01	0.16	3.63E−01		*IPO7*
chr12:10541381–10541435	0.78	0.44	0.67	0.34	2.29E−02	0.11	5.75E−01	−0.23	1.33E−01		*KLRK1*
chr12:11877800–11877835	0.40	0.75	0.58	−0.35	1.36E−02	−0.18	5.75E−01	0.17	5.63E−01		*ETV6*
chr12:49760043–49760055	0.17	0.55	0.17	−0.38	4.46E−02	0.00	1.00E+00	0.38	7.62E−02		*SPATS2*
chr13:42086755–42086780	0.45	0.88	0.29	−0.43	7.51E−04	0.16	6.86E−01	0.59	3.75E−02		
chr13:79233596–79233703	0.24	0.04	0.40	0.20	1.04E−03	−0.15	9.20E−01	−0.36	9.36E−02		*OBI1*
chr13:79234435–79234484	0.57	0.22	0.44	0.34	2.02E−02	0.13	8.52E−01	−0.22	2.31E−01	cg21838488	
chr14:100991389–100991402	0.73	0.27	0.50	0.45	3.10E−05	0.23	6.43E−01	−0.23	4.18E−01		*WDR25*
chr14:31160006–31160287	0.49	0.85	0.89	−0.36	1.17E−04	−0.40	3.45E−01	−0.04	7.70E−01		*SCFD1*
chr14:32932673–32932707	0.81	0.34	0.83	0.47	2.48E−03	−0.01	1.00E+00	−0.48	4.39E−02		*AKAP6*
chr14:62585760–62585806	0.49	0.75	0.51	−0.26	4.87E−02	−0.03	9.20E−01	0.24	1.89E−01		*LINC00643*
chr14:78023547–78024047	0.85	0.51	0.80	0.34	3.43E−02	0.05	7.60E−01	−0.28	7.62E−02	cg03678595	*SPTLC2*
chr15:40348298–40348308	0.47	0.81	0.74	−0.35	2.74E−02	−0.27	4.17E−01	0.08	6.02E−01	cg14482712	*SRP14-AS1*
chr15:61907419–61907481	0.32	0.54	0.56	−0.22	3.25E−02	−0.24	3.45E−01	−0.02	9.48E−01		
chr15:66461530–66461578	0.55	0.86	0.77	−0.31	3.47E−04	−0.22	5.75E−01	0.10	8.53E−01		*MEGF11*
chr15:70387017–70387094	0.50	0.84	0.53	−0.34	3.53E−02	−0.03	8.52E−01	0.31	4.57E−02		*TLE3*
chr16:83560245–83560262	0.45	0.78	0.74	−0.33	4.93E−03	−0.29	3.45E−01	0.04	7.53E−01		*CDH13*
chr16:85137169–85137403	0.47	0.10	0.63	0.37	1.70E−05	−0.16	6.43E−01	−0.53	7.62E−02		*CIBAR2*
chr16:85137527–85137561	0.47	0.16	0.64	0.31	3.99E−02	−0.17	5.75E−01	−0.48	5.84E−02	cg11407606	*CIBAR2*
chr16:87402123–87402150	0.72	0.28	0.31	0.44	1.41E−03	0.41	3.45E−01	−0.04	7.53E−01	cg21533743	*FBXO31*
chr16:89628537–89628548	0.23	0.44	0.42	−0.21	2.41E−02	−0.19	6.43E−01	0.03	9.42E−01		*RPL13*
chr17:10056902–10057753	0.56	0.86	0.79	−0.30	1.86E−03	−0.23	4.92E−01	0.07	2.62E−01		*GAS7*
chr17:13852922–13852978	0.25	0.67	0.75	−0.42	5.02E−03	−0.50	3.45E−01	−0.08	7.45E−01		
chr17:36366120–36366293	0.76	0.57	0.69	0.20	1.44E−04	0.08	3.73E−01	−0.12	5.84E−02		*TBC1D3*
chr17:3977443–3977460	0.40	0.10	0.21	0.30	1.87E−02	0.19	6.43E−01	−0.12	1.72E−01		*ZZEF1*
chr17:53622508–53622529	0.38	0.73	0.63	−0.35	2.52E−02	−0.25	3.73E−01	0.10	4.64E−01		
chr17:58836032–58836042	0.03	0.82	0.15	−0.79	2.12E−06	−0.12	6.43E−01	0.67	4.95E−02		*BCAS3*
chr17:8646352–8646375	0.48	0.88	0.55	−0.40	3.67E−03	−0.07	8.52E−01	0.33	5.84E−02		*CCDC42*
chr17:9872964–9873016	0.70	0.90	0.72	−0.20	3.78E−02	−0.03	9.20E−01	0.18	7.62E−02		*GAS7*
chr17:9964598–9964795	0.58	0.84	0.77	−0.26	1.17E−02	−0.19	3.73E−01	0.08	5.09E−01		*GAS7*
chr18:57842408–57842698	0.46	0.12	0.67	0.34	5.30E−03	−0.21	3.73E−01	−0.55	3.04E−02		
chr19:12877235–12877296	0.58	0.10	0.31	0.48	2.09E−06	0.27	3.73E−01	−0.21	1.57E−01		*HOOK2*
chr19:37266989–37267026	0.55	0.87	0.68	−0.32	2.29E−02	−0.12	6.86E−01	0.19	2.96E−01		
chr19:45224382–45224442	0.61	0.23	0.39	0.37	2.52E−02	0.21	3.45E−01	−0.16	1.72E−01		
chr19:47894453–47894605	0.55	0.19	0.42	0.36	5.30E−03	0.13	4.17E−01	−0.23	7.97E−02		
chr19:7537300–7537423	0.49	0.17	0.37	0.31	3.46E−03	0.12	6.43E−01	−0.20	6.82E−02		
chr2:111704737–111704759	0.35	0.67	0.53	−0.33	3.79E−02	−0.19	4.17E−01	0.14	4.64E−01		*ACOXL*
chr2:174013389–174013418	0.55	0.86	0.84	−0.30	1.21E−02	−0.29	3.45E−01	0.02	9.01E−01		*MAP3K20*
chr2:238439479–238439609	0.61	0.18	0.61	0.42	6.23E−08	−0.01	1.00E+00	−0.43	3.04E−02		*MLPH*
chr2:48746791–48746890	0.65	0.93	0.89	−0.28	1.64E−04	−0.24	3.45E−01	0.04	8.53E−01		
chr2:86448198–86448322	0.08	0.46	0.07	−0.38	3.33E−02	0.01	9.20E−01	0.39	5.84E−02		*REEP1*
chr20:24642759–24642792	0.42	0.69	0.58	−0.27	3.51E−02	−0.16	5.75E−01	0.11	4.18E−01		*SYNDIG1*
chr20:57796902–57796976	0.36	0.70	0.61	−0.34	2.04E−02	−0.24	3.45E−01	0.09	5.48E−01		*ZNF831*
chr21:10782899–10782914	0.34	0.65	0.31	−0.31	1.22E−02	0.03	8.52E−01	0.34	4.57E−02		
chr21:45969479–45969498	0.49	0.16	0.45	0.33	4.93E−03	0.04	9.20E−01	−0.29	5.97E−02		*TSPEAR*
chr22:18579345–18579463	0.46	0.75	0.47	−0.29	4.68E−02	−0.01	1.00E+00	0.28	1.16E−01		
chr22:25875099–25875361	0.83	0.43	0.88	0.40	8.64E−04	−0.05	7.60E−01	−0.45	3.04E−02		
chr22:49852930–49852978	0.47	0.75	0.74	−0.28	2.67E−02	−0.26	4.92E−01	0.01	8.61E−01		*C22orf34*
chr22:49854103–49854290	0.47	0.79	0.71	−0.32	2.36E−03	−0.25	3.45E−01	0.08	4.64E−01		*C22orf34*
chr22:49868167–49868239	0.62	0.83	0.82	−0.21	2.86E−02	−0.20	3.73E−01	0.01	8.31E−01		*C22orf34*
chr22:49899480–49899619	0.56	0.82	0.86	−0.26	3.24E−02	−0.31	3.45E−01	−0.05	8.53E−01		*C22orf34*
chr22:49913051–49913121	0.55	0.88	0.83	−0.33	2.58E−05	−0.27	3.45E−01	0.06	4.32E−01		*C22orf34*
chr22:49913486–49913581	0.25	0.57	0.45	−0.33	1.31E−03	−0.20	4.17E−01	0.12	4.61E−01		*C22orf34*
chr22:49915280–49915358	0.34	0.76	0.70	−0.42	2.98E−03	−0.36	3.45E−01	0.06	8.61E−01	cg21377026	*C22orf34*
chr22:49938239–49938304	0.60	0.84	0.86	−0.24	5.30E−03	−0.26	3.45E−01	−0.02	8.38E−01		*C22orf34*
chr3:101954229–101954837	0.61	0.16	0.68	0.45	2.03E−04	−0.07	6.86E−01	−0.52	3.28E−02	cg09183220	
chr3:187491756–187491972	0.54	0.15	0.44	0.39	2.46E−04	0.10	6.86E−01	−0.29	7.46E−02		
chr4:100176744–100176831	0.35	0.77	0.79	−0.43	1.29E−04	−0.45	3.45E−01	−0.02	9.48E−01		*ADH6*
chr4:144811744–144811754	0.34	0.73	0.55	−0.39	2.05E−02	−0.22	4.92E−01	0.18	2.96E−01		*GYPE*
chr4:39168987–39169269	0.51	0.17	0.46	0.34	1.82E−03	0.05	8.52E−01	−0.29	4.57E−02		*WDR19*
chr4:70691044–70691128	0.20	0.58	0.33	−0.38	8.76E−03	−0.13	4.92E−01	0.25	1.59E−01		
chr5:150507603–150507621	0.39	0.78	0.63	−0.39	1.08E−02	−0.25	4.17E−01	0.15	2.62E−01		*ANXA6*
chr5:169821447–169821476	0.43	0.78	0.78	−0.35	3.79E−02	−0.35	3.45E−01	0.00	8.38E−01		*KCNIP1*
chr5:40286409–40286502	0.61	0.25	0.84	0.36	9.99E−03	−0.24	4.17E−01	−0.60	3.04E−02		
chr5:76651093–76651280	0.59	0.28	0.47	0.31	3.18E−02	0.12	6.86E−01	−0.20	1.97E−01		*PDE8B*
chr6:112164213–112164419	0.39	0.75	0.39	−0.36	3.76E−02	−0.01	7.60E−01	0.35	4.57E−02		*FYN*
chr6:147839678–147839773	0.46	0.17	0.48	0.28	2.28E−02	−0.02	9.20E−01	−0.31	7.62E−02		*SAMD5*
chr6:148663571–148663585	0.19	0.04	0.06	0.15	3.81E−04	0.13	4.92E−01	−0.02	3.35E−01	cg06081609	*SASH1*
chr6:159703533–159703779	0.08	0.59	0.47	−0.51	9.22E−04	−0.39	3.45E−01	0.12	4.61E−01		
chr7:105766161–105766466	0.73	0.19	0.40	0.54	2.45E−03	0.33	3.73E−01	−0.21	1.89E−01		
chr7:55835533–55835721	0.54	0.90	0.80	−0.36	8.36E−06	−0.26	3.45E−01	0.10	1.49E−01		
chr7:55836053–55836330	0.40	0.91	0.73	−0.51	5.75E−10	−0.33	3.45E−01	0.18	9.15E−02		
chr7:55837127–55837253	0.29	0.89	0.70	−0.60	3.35E−11	−0.41	3.45E−01	0.19	1.70E−01		
chr7:55842459–55842504	0.32	0.88	0.46	−0.56	5.75E−10	−0.14	6.86E−01	0.42	7.62E−02		
chr7:90895629–90895657	0.67	0.93	0.74	−0.25	2.98E−03	−0.06	7.60E−01	0.19	7.78E−02		*FZD1*
chr8:136696891–136696912	0.25	0.64	0.45	−0.40	2.04E−02	−0.20	5.75E−01	0.19	3.76E−01		
chr8:141952722–141952746	0.60	0.89	0.77	−0.29	2.24E−04	−0.17	4.92E−01	0.12	5.09E−01		*PTK2*
chr8:144437504–144437509	0.34	0.72	0.47	−0.38	2.04E−02	−0.13	6.86E−01	0.26	1.66E−01	cg22123784	*TOP1MT*
chr8:146252130–146252142	0.14	0.42	0.37	−0.28	3.38E−02	−0.23	3.45E−01	0.05	7.88E−01		
chr8:41594071–41594093	0.38	0.73	0.58	−0.35	4.39E−02	−0.19	6.43E−01	0.16	2.62E−01		*ANK1*
chr9:27340169–27340184	0.48	0.83	0.84	−0.35	4.86E−04	−0.36	3.45E−01	−0.01	9.63E−01		*MOB3B*

*Note*: *, genes whose promoters and gene body regions overlap with Pop-CMRs. AML, average methylation level (defined as the mean methylation level of correlated CpG sites within the CMR). Statistical significance was defined as Benjamini–Hochberg FDR < 0.05. Previously reported population-specific CpGs, including 110,313 CpGs, were collected from previous array-based studies. These CpGs are listed in Table S10. CMR, co-methylated region; Pop-CMR, population-specific CMR; chr, chromosome; FDR, false discovery rate; EUR, European; AFR, African; EAS, East Asian.

We further validated these Pop-CMRs using the combined HM450K dataset described above, which includes 326 LCL samples (EUR *n* = 157, AFR *n* = 169) (Table S3) [12,14,30]. This analysis aimed to determine whether the array CpGs located within Pop-CMRs (*n* = 7) were enriched for population-specific CpGs (|AML difference| ≥ 0.1 between populations and Mann–Whitney U test FDR < 0.05) [33]. We found that 57.1% (4/7) of the CpGs within Pop-CMRs were population-specific and showed a consistent direction of change between the AFR and EUR populations as observed in the WGBS data (Table S7). Compared to a random selection of seven CpGs from all array CpGs (*n* = 413,012) or from the CpGs covered by all CMRs (*n* = 3936), the CpGs in Pop-CMRs were 111.0-fold and 17.4-fold more likely to be population-specific, respectively (1000 bootstrap replicates, both *P* < 0.001; Figure 2F). Notably, all seven CpGs have previously been reported as population-specific in array-based studies [9,16,35] (Table S8). These results confirm that even a small, independently profiled subset of Pop-CMRs is highly enriched for reproducible population-specific methylation differences, reinforcing the biological validity of the Pop-CMRs identified in the WGBS data.

Additionally, we found that CpG count and density do not, or very weakly, influence the discovery of Pop-CMRs. The CpG count between Pop-CMRs and random CMR sets did not differ significantly (3.89 *vs*. 3.67, respectively, 1,000,000 bootstrap replicates, *P* = 0.095). Moreover, there was no significant enrichment or depletion of Pop-CMRs in CpG-rich regions (*e.g.*, CpG islands) or CpG-poor regions (*e.g.*, introns) compared to random sets of CMRs (1,000,000 bootstrap replicates, all FDR > 0.05; Table S9), whereas enrichment in introns and depletion in CpG islands were observed (both *P* < 0.05; Figure S4). This result aligns with the findings obtained using the EPIC array [9], but contrasts with those using the HM450K array, which showed that population-specific DNAm sites were significantly depleted in CpG islands [12]. These findings suggest that leveraging a broader CpG coverage by, for example, using high-coverage microarray or WGBS, may yield more precise insights into the biological characteristics of population-specific DNAm.

### The majority of population-specific DNAm patterns were exclusively identified in our WGBS-based analysis compared to previous array-based studies

Although previous analyses have identified population-specific DNAm sites using arrays, primarily the HM450K array, one expected advantage of this study is the discovery of additional population-specific sites due to the comparatively high number of CpGs measured in WGBS. To highlight this advantage, we examined the overlap and discrepancies between Pop-CMRs and previous array-based findings, including the HM27K, HM450K, and EPIC arrays. A total of 110,510 population-specific CpGs, primarily between EUR and AFR but also other populations, were collected from previous array-based investigations regardless of tissue type [9–17] (Table S10). Only 10 Pop-CMRs were found to overlap with these population-specific CpGs (Table 1). That is, DNAm patterns in 91 Pop-CMRs were uniquely discovered to be associated with population differences in this study, highlighting the major benefit of our whole-genome DNAm analysis in detecting such alterations, even with a limited sample size.

Comparing Pop-CMRs to all CpGs measurable by arrays (HM27K, HM450K, and EPIC), only 15 of 101 (15%) were quantifiable by at least one array (*i.e*., Pop-CMRs covering at least one array CpG) (Figure 2G). Of those, five Pop-CMRs overlapped with array CpGs that were not previously been recognized as population-specific. Three of these were excluded due to known technical artifacts in array probes, including cross-reactivity and the influence of single nucleotide polymorphisms (SNPs) on the measured CpGs (*i.e*., probe adjacent to a SNP) [36–38] (Figure 2G; Table S11). In WGBS data, sequencing reads aligned uniquely to the human reference genome were used to measure DNAm for these CpG sites, aided by their longer read length (length > 100 bp) compared to the shorter array probe sequence length (length = 50 bp). Unlike array-based DNAm measurements, which are affected by adjacent SNPs due to their impact on probe mappability [37,38], WGBS reads are generated by independent sequencing chemistry. The two remaining Pop-CMRs, which overlapped with array CpGs that were not previously identified as population-specific, may have been missed due to the limited number of studies using the EPIC array. Only one study has examined CpG DNAm differences between EUR and AFR individuals using the EPIC array [9]. However, multiple adjacent CpGs (distance < 15 kb) have been identified as population-specific CpGs in previous array-based investigations (Table S12). These results indicate that our whole-genome DNAm analysis offers key advantages over previous array-based analyses, such as avoiding issues related to probe cross-reactivity and SNP interference, providing greater power in detecting population-specific DNAm alterations.

Comparing genes associated with Pop-CMRs to those identified in population-specific EWAS findings is of particular interest, as multiple CpGs in DMRs could regulate the same genes. To investigate this, we examined the overlap between the genes containing population-specific CpGs discovered in previous array-based analyses and the 54 genes overlapping with Pop-CMRs (Table 1). Four genes (*CCDC42*, *GYPE*, *MAP3K20*, and *OBI1*) uniquely overlapped with Pop-CMRs, with three of these (*GYPE*, *MAP3K20*, and *OBI1*) being identified exclusively due to the absence of array probes targeting CpGs within these genes. These four genes have been previously associated with diseases that vary in prevalence and incidence across populations, such as type 2 diabetes mellitus [39], malaria infection [40], Alzheimer’s disease [41], and large artery stroke [42], suggesting potential biological roles for genes exclusively overlapping with Pop-CMRs. Taken together, our analysis identified a distinct set of population-specific and gene-associated DNAm patterns linked to several metabolic and infectious diseases.

### Pop-CMRs were enriched in genes relevant to metabolism and infection

Given the known role of DNAm in transcription regulation [9,43], Pop-CMRs may be associated with population-specific transcription. Associations between Pop-CMR methylation and gene expression were assessed using matched microarray-based transcriptomic and WGBS data from nine AFR and five EUR LCL samples (GEO: GSE23120, GSE11582, and GSE49628) [28,44,45]. Linear regression analysis across these LCL samples showed that the expression levels of four genes (*DSTYK*, *GAS7*, *SASH1*, and *TLE3*) were associated with Pop-CMR methylation levels after adjustment for population and sex (*r* > 0.3 and *P* < 0.05) (Table S13). Subsequently, the differential expression of the 54 genes mapped to Pop-CMRs was tested using Mann–Whitney U on transcriptomic profiles from 149 AFR and 156 EUR subjects (GEO: GSE23120, GSE11582, and GSE49628) [28,44,45]. This dataset included 14 transcriptomic profiles with matched DNAm datasets, as well as 291 additional transcriptomic profiles. Two genes (*GAS7* and *SASH1*) were differentially expressed between EUR and AFR populations with FDR < 0.05 (Figure S5). These findings suggest that population-specific methylation may play a regulatory role in population-specific gene expression.

Given the limited availability of matched transcriptomic and DNAm data (*n* = 14), our understanding of the biological relevance of genes associated with Pop-CMRs may be constrained. To address this, we identified relevant genes in two ways: (1) genes whose promoters or gene bodies overlap with Pop-CMRs, and (2) genes whose transcription is significantly associated with variation in DNAm at any CpGs within a Pop-CMR using the iMETHYL database, which includes WGBS and whole-transcriptome sequencing data from peripheral blood mononuclear cells and blood cell types from Japanese individuals [46].

By overlaying Pop-CMRs on promoters or gene body regions, 54 unique genes were linked to 64 Pop-CMRs (Table 1). Functional enrichment analysis showed significant enrichment of these genes in 312 Gene Ontology (GO) terms, 1 Kyoto Encyclopedia of Genes and Genomes (KEGG) pathway, and 5 Reactome pathways (FDR < 0.05) with at least 5 tested genes per GO term/pathway (Table S14). The 312 GO terms were further classified based on ancestor GO terms [47], where the top 3 specific terms were metabolism process, biosynthesis process, and binding (Figure 2H). Consistent with the evolution of the B-cell immune response in humans [48], we observed enrichment in infection-related biological processes, such as response to stimulus (GO:0050896; FDR = 1.02 × 10^−6^) and defense response (GO:0006952; FDR = 0.047) (Table S14).

Using the iMETHYL database, the expression levels of 48 genes were found to be associated with 30 Pop-CMRs (Table S15), with 10 of these genes also identified in the aforementioned GO enrichment analysis (Figure 2H). These 48 genes were significantly enriched in 208 GO terms, 1 KEGG pathway, and 4 Reactome pathways, under the same thresholds (FDR < 0.05; ≥ 5 genes per GO term/pathway) (Table S16). The top 3 ancestor GO terms were metabolism process, biosynthesis process, and cellular component (Figure 2H). Additional enriched terms/pathways included immune-related biological processes, such as response to stimulus (GO:0050896; FDR = 1.96 × 10^−5^), immune response (GO:0006955; FDR = 1.78 × 10^−4^), regulation of immune system process (GO:0002682; FDR = 2.19 × 10^−4^), defense response (GO:0006952; FDR = 4.72 × 10^−4^), leukocyte activation (GO:0045321; FDR = 4.51 × 10^−3^), and infectious disease (REAC:R-HSA-5663205; FDR = 0.009) (Table S16). Similar enrichment patterns for both gene sets suggest potential roles for population-specific DNAm in these biological processes, particularly metabolism and biosynthesis.

### Increased between-population DNAm differences in Pop-CMRs were linked to nearby genetic variants

**Figure 3:**
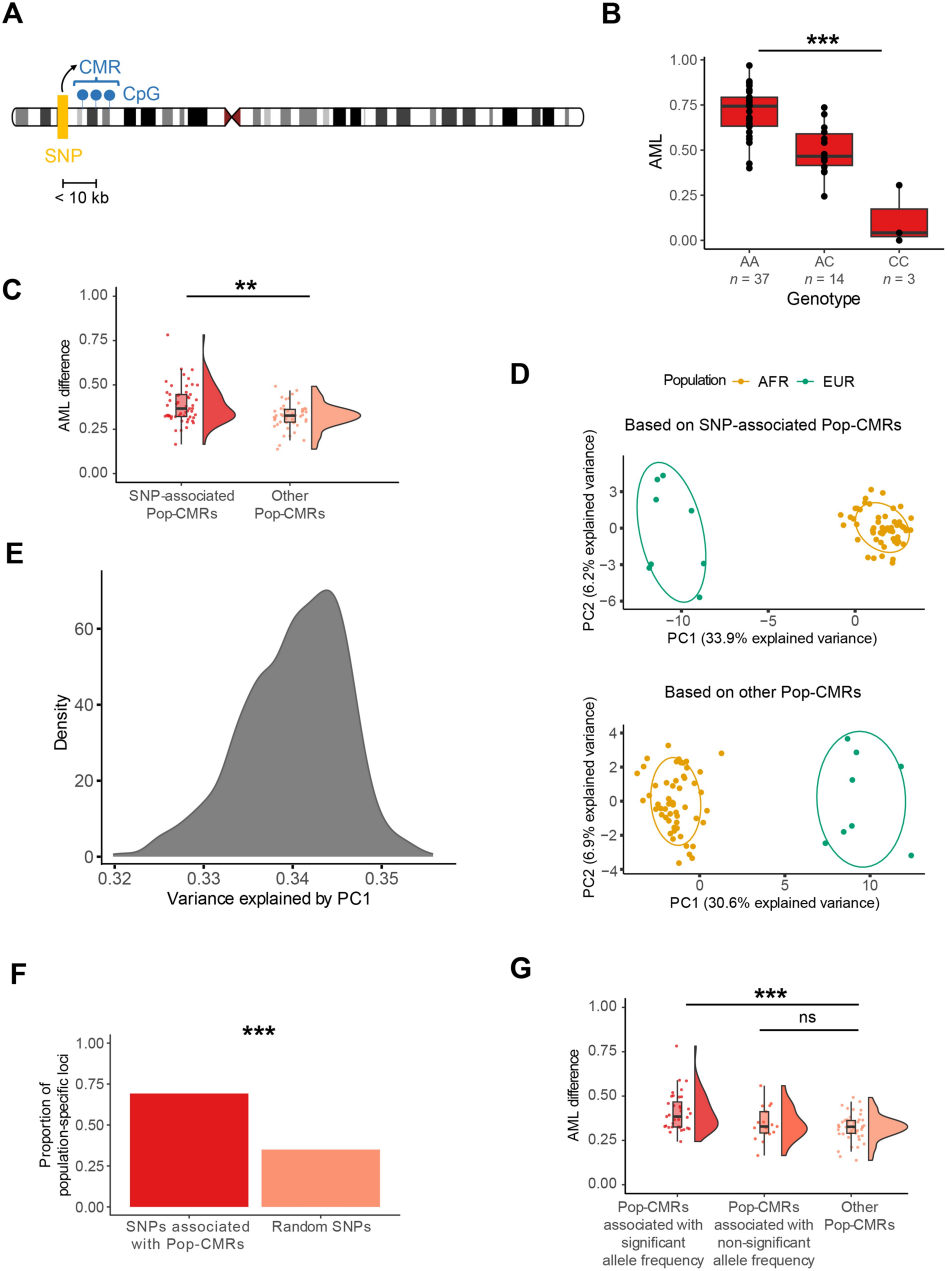
Interplay between Pop-CMRs and genetic variation **A**. Schematic illustration of potential local genetic effects on CMR methylation with SNP–CMR distance < 10 kb [12,51]. **B**. Box plot showing AMLs at the Pop-CMR (chr10:54504049–54504586) stratified by genotype at SNP rs996233, suggesting significant association between genetic variation and DNAm of Pop-CMR. ***, FDR < 0.001. **C**. Box plot summarizing increased AML variability between EUR and AFR populations in SNP-associated Pop-CMRs using the absolute values of AML differences. **, 0.001 ≤ *P* < 0.01. **D**. PCA of AMLs at 53 SNP-associated Pop-CMRs (top) and 48 SNP-independent Pop-CMRs (bottom) across LCL WGBS samples. Each point represents an individual, colored by population. Clustering reflects population-specific DNAm patterns. **E**. Density plot showing the proportion of variance explained by PC1 across 1000 bootstrap replicates of randomly selected SNP-associated Pop-CMRs. **F**. Proportion of population-specific loci among SNPs associated with Pop-CMRs compared to randomly sampled SNPs. Population-specific loci were defined as SNPs with |AF difference| > 0.1 between EUR and AFR populations and FDR < 0.05 (Fisher’s exact test). Random sampling was repeated 1000 times. ***, *P* < 0.001 (1000 bootstrap replicates). **G**. Box plot summarizing AML variability between EUR and AFR populations in subgroups of SNP-associated Pop-CMRs compared to SNP-independent Pop-CMRs. ***, FDR < 0.001; ns, not significant. SNP, single nucleotide polymorphism; PCA, principal component analysis; AF, allele frequency.

DNAm levels at more than half of the population-specific CpGs discovered in previous studies (55.4%–70.2%) have been associated with genetic variants [9–11,49]. However, these previous studies primarily focused on CpGs measured by arrays. As the majority of Pop-CMRs identified herein are not measured on any array, the extent to which their population specificity is associated with genetic variation is largely unclear. To address this gap, we retrieved whole-genome sequencing (WGS) genotype data from the 1000 Genomes Project Phase 3 (Table S1) [50] for the 54 AFR samples. Associations between each Pop-CMR and *cis*-acting SNPs (within a 10-kb window [12,51]) were evaluated using linear modeling with adjustment for sex (**Figure 3**A).

We found that 53 of 101 Pop-CMRs (52.5%) were associated with 707 SNPs (FDR < 0.05), consistent with previous array-based findings [9–11] (Table S17). Figure 3B illustrates an example of how DNAm in a Pop-CMR (chr10:54504049–54504586) varies by genotype at SNP rs996233. Given that multiple SNPs can be associated with the same CMR, only the most significantly associated SNP (*i.e.*, the one with the lowest *P* value) was used in subsequent analyses (**Table 2**; Figure S6). These SNPs explained an average of 30.6% (range: 14.7%–64.4%) of the Pop-CMR DNAm variance, lower than that reported in a previous array-based study in monocytes (58%) [9] but slightly higher than that reported in a previous array-based study in LCLs (26%) [11]. These findings suggest that the association between DNAm and population specificity may be influenced by sequence variants, particularly considering only *cis-*SNPs.

**Table 2:** SNPs associated with Pop-CMRs and their AF differences across three human populations

SNP	Pop-CMR	SNP–Pop-CMR association		AF			EUR *vs.* AFR		EUR *vs.* EAS		AFR *vs.* EAS	
		Beta	FDR	EUR	AFR	EAS	AF difference	FDR	AF difference	FDR	AF difference	FDR
rs12752777	chr1:105416732–105416911	0.19	7.92E−06	0.28	0.66	0.49	−0.38	1.70E−27	−0.21	4.86E−10	0.17	2.53E−05
rs777724917	chr1:1329689–1329749	0.23	4.86E−03	0.82	0.17	0.02	0.65	1.73E−84	0.79	1.87E−161	0.15	1.05E−29
rs869217559	chr1:205141231–205141245	−0.18	1.71E−02	0.54	0.17	0.47	0.38	1.09E−41	0.07	1.12E−01	−0.31	1.72E−31
chr1:36249688	chr1:36724301–36724326	−0.41	1.58E−02	0.18	0.03	0.12	0.15	2.77E−27	0.06	2.51E−03	−0.09	9.14E−14
rs373437560	chr1:570123–570221	0.34	1.05E−02	0.14	0.03	0.00	0.12	1.63E−22	0.14	1.40E−35	0.02	7.37E−06
rs9425029	chr1:71013831–71013852	−0.15	3.89E−02	0.30	0.64	0.57	−0.34	4.20E−22	−0.26	1.55E−13	0.07	8.59E−02
rs1592133162	chr10:128918479–128918639	−0.19	1.21E−03	0.14	0.70	0.16	−0.56	2.25E−72	−0.03	2.29E−01	0.54	1.95E−62
rs12573118	chr10:54504049–54504586	−0.26	1.15E−09	0.57	0.17	0.59	0.39	1.07E−43	−0.02	7.30E−01	−0.41	1.56E−46
rs3758640	chr11:33910910–33910955	0.09	2.87E−02	0.36	0.38	0.33	−0.01	6.55E−01	0.03	3.98E−01	0.05	1.24E−01
rs498045	chr11:66362800–66362849	−0.45	5.68E−08	0.51	0.05	0.38	0.46	2.07E−94	0.13	4.47E−04	−0.33	4.83E−64
rs2137995	chr11:82169987–82170030	−0.27	4.35E−05	0.73	0.21	0.32	0.53	8.30E−58	0.42	9.91E−26	−0.11	5.34E−06
rs12801924	chr11:9419206–9419271	−0.24	1.31E−03	0.59	0.17	0.20	0.41	2.06E−46	0.39	7.79E−33	−0.03	1.97E−01
rs750497144	chr12:49760043–49760055	−0.18	4.87E−02	0.17	0.73	0.01	−0.56	2.75E−64	0.16	8.15E−35	0.71	1.42E−167
rs9594561	chr13:42086755–42086780	−0.59	3.15E−04	0.06	0.03	0.04	0.03	1.37E−04	0.02	7.30E−02	−0.01	8.55E−02
rs7149947	chr14:32932673–32932707	0.29	6.30E−06	0.14	0.73	0.39	−0.59	2.18E−77	−0.26	1.23E−23	0.33	3.14E−17
rs192210212	chr15:70387017–70387094	−0.17	4.44E−02	0.06	0.06	0.13	0.00	1.00E+00	−0.07	7.24E−07	−0.07	4.41E−08
rs6564101	chr16:85137169–85137403	0.17	1.28E−02	0.58	0.11	0.66	0.46	2.43E−67	−0.09	7.66E−02	−0.55	6.26E−83
rs75835349	chr16:89628537–89628548	−0.38	9.40E−03	0.00	0.11	0.00	−0.11	2.21E−35	0.00	1.00E+00	0.11	4.00E−35
rs59778642	chr17:9964598–9964795	−0.15	2.30E−02	0.00	0.06	0.00	−0.06	4.67E−20	0.00	1.00E+00	0.06	7.67E−20
chr17:10154917	chr17:10056902–10057753	−0.19	3.90E−02	0.10	0.11	0.06	−0.01	6.17E−01	0.04	2.94E−03	0.05	1.47E−04
rs12943919	chr17:13852922–13852978	−0.28	8.09E−05	0.69	0.25	0.41	0.45	1.58E−40	0.28	4.33E−11	−0.17	1.53E−09
rs76210116	chr17:3977443 − 3977460	0.45	4.50E−04	0.00	0.09	0.00	−0.09	8.62E−30	0.00	1.00E+00	0.09	1.15E−29
rs4273103	chr17:53622508 − 53622529	−0.25	1.62E−02	0.68	0.10	0.40	0.58	2.07E−94	0.28	1.55E−11	−0.30	5.42E−41
rs11658417	chr17:58836032–58836042	−0.42	2.39E−10	0.10	0.81	0.02	−0.70	2.06E−105	0.09	2.31E−15	0.79	1.26E−175
rs12949056	chr17:8646352–8646375	−0.25	1.45E−02	0.29	0.09	0.18	0.21	7.80E−28	0.12	1.92E−06	−0.09	2.23E−08
rs2051312	chr18:57842408–57842698	0.28	1.45E−10	0.56	0.17	0.76	0.39	3.86E−44	−0.19	6.39E−05	−0.59	1.51E−74
rs60488314	chr19:12877235–12877296	0.36	1.31E−03	0.00	0.02	0.00	−0.02	4.92E−06	0.00	1.00E+00	0.02	5.59E−06
rs1188390220	chr19:37266989–37267026	−0.40	1.05E−02	0.00	0.01	0.00	−0.01	3.61E−02	0.00	5.91E−01	0.01	2.18E−03
rs148668754	chr19:45224382–45224442	0.29	9.24E−04	0.29	0.17	0.15	0.12	1.28E−07	0.14	7.27E−10	0.03	1.73E−01
rs729386	chr2:111704737–111704759	−0.12	1.11E−02	0.31	0.41	0.24	−0.10	4.65E−04	0.07	1.51E−02	0.17	7.06E−10
rs749579	chr2:86448198–86448322	−0.27	2.38E−06	0.13	0.50	0.24	−0.37	8.60E−45	−0.11	2.08E−07	0.26	6.68E−18
rs468784	chr22:18579345–18579463	−0.16	1.05E−02	0.67	0.28	0.34	0.39	9.04E−30	0.33	5.10E−17	−0.06	3.41E−02
rs5770463	chr22:49852930–49852978	−0.12	4.07E−02	0.24	0.45	0.44	−0.21	3.87E−13	−0.20	1.84E−10	0.01	7.20E−01
rs73444431	chr22:49915280–49915358	−0.48	8.92E−03	0.00	0.03	0.00	−0.03	3.27E−09	0.00	1.00E+00	0.03	3.84E−09
rs1601790982	chr22:49913051–49913121	−0.18	1.37E−02	0.01	0.06	0.00	−0.05	9.76E−09	0.01	7.69E−04	0.06	1.50E−18
rs1157681679	chr22:49913486–49913581	−0.41	2.31E−02	0.00	0.01	0.00	0.00	1.42E−01	0.00	5.60E−01	0.00	7.88E−01
rs67733349	chr3:101954229–101954837	0.22	1.21E−09	0.14	0.65	0.00	−0.51	2.43E−64	0.14	1.90E−36	0.65	3.97E−167
rs1851683	chr3:187491756–187491972	0.25	9.00E−07	0.67	0.19	0.46	0.47	5.88E−52	0.20	2.45E−06	−0.27	3.02E−23
rs1220721	chr4:70691044–70691128	−0.18	1.98E−02	0.15	0.77	0.51	−0.62	1.76E−78	−0.36	2.43E−35	0.26	2.23E−09
chr4:99250947	chr4:100176744–100176831	−0.31	1.31E−03	0.40	0.06	0.02	0.34	1.85E−64	0.38	2.75E−75	0.03	3.38E−04
rs4957263	chr5:40286409–40286502	0.16	1.32E−03	0.49	0.19	0.69	0.30	1.90E−27	−0.21	4.00E−06	−0.50	2.32E−56
rs623003	chr6:147839678–147839773	0.14	8.17E−03	0.67	0.17	0.52	0.50	4.55E−60	0.15	7.57E−04	−0.35	1.54E−36
rs1477911658	chr6:148663571–148663585	0.05	8.25E−04	0.06	0.07	0.03	0.00	8.16E−01	0.03	5.33E−03	0.03	8.61E−04
rs543547	chr6:159703533–159703779	−0.26	9.00E−07	0.16	0.64	0.36	−0.48	2.17E−56	−0.21	5.25E−16	0.27	9.14E−14
rs6966151	chr7:105766161–105766466	0.35	1.15E−09	0.17	0.74	0.27	−0.57	3.93E−67	−0.10	3.54E−05	0.47	5.71E−39
rs12386672	chr7:55836053–55836330	−0.24	3.71E−05	0.77	0.10	0.36	0.67	1.43E−113	0.41	1.70E−22	−0.26	3.40E−35
rs12386672	chr7:55835533–55835721	−0.15	4.65E−02	0.77	0.10	0.36	0.67	1.43E−113	0.41	1.70E−22	−0.26	3.40E−35
rs11238378	chr7:55837127–55837253	−0.26	3.16E−06	0.77	0.10	0.36	0.67	2.87E−112	0.41	2.15E−22	−0.26	1.80E−34
rs187381548	chr7:55842459–55842504	−0.34	2.04E−08	0.02	0.03	0.00	−0.01	1.00E−01	0.02	5.57E−05	0.03	3.84E−09
rs535084233	chr7:90895629–90895657	−0.32	1.41E−04	0.00	0.00	0.00	0.00	5.52E−01	0.00	1.00E+00	0.00	5.29E−01
rs73377504	chr8:136696891–136696912	0.30	2.26E−02	0.00	0.05	0.00	−0.05	2.49E−15	0.00	5.91E−01	0.05	9.68E−17
rs10672408	chr8:141952722–141952746	−0.12	1.05E−02	0.55	0.12	0.40	0.43	2.50E−60	0.15	1.49E−04	−0.28	2.54E−34
rs11984612	chr8:41594071–41594093	−0.35	2.55E−02	0.00	0.02	0.00	−0.02	9.18E−06	0.00	1.00E+00	0.02	1.01E−05

*Note*: *, for each Pop-CMR, only the most significantly associated SNP (*i.e.*, the one with the lowest P value) is shown, given that multiple SNPs can be associated with the same CMR. AF differences for SNPs were calculated between any two of the three populations. Statistical significance was defined as Benjamini–Hochberg FDR < 0.05. SNP, single nucleotide polymorphism; AF, allele frequency.

It is important to study how genetic variants influence the population specificity of DNAm, as increased DNAm differences between populations influenced by genetic variation may indicate a significant genetic contribution. We found that between-population DNAm differences were significantly larger for SNP-associated Pop-CMRs (*n* = 53) than those for SNP-independent Pop-CMRs (*n* = 48) (mean: 0.39 *vs*. 0.32, respectively; Mann–Whitney U test, *P* = 0.002) (Figure 3C). Furthermore, PCA using the SNP-associated Pop-CMRs clearly distinguished between the two populations, with PC1 accounting for 33.9% of the total variance (Figure 3D). Although the SNP-independent Pop-CMRs could also distinguish between the two populations, they explained slightly less variance (30.6%). To address the concern that the difference in PC-explained variance may be due to the unbalanced number of SNPs in the two sets, 1000 bootstrap replicates were performed using SNP-independent Pop-CMRs and the same number of randomly selected SNP-associated Pop-CMRs. The PC1 for the SNP-associated Pop-CMRs exhibited a significantly larger explained variance than that for the SNP-independent Pop-CMRs [34.0% (range: 32.0%–35.6%) *vs*. 30.6%, *P* < 0.001) (Figure 3E). As the SNP-independent Pop-CMRs defined here may be associated with *trans*-SNPs or other genetic variations (such as copy number variants), these observations could become even more pronounced when broader genetic effects are considered. Taken together, our results suggest that genetic variation is associated with increased between-population DNAm differences in Pop-CMRs, and indicates a critical role of genetic variation in shaping population-specific DNAm patterns.

### Allele frequency differences may explain the role of genetic variants in population-specific DNAm

We next explored whether the genetic influence on population specificity of DNAm could be attributed to differences in allele frequency (AF). First, we tested whether the genetic variants associated with population-specific DNAm exhibited varied AFs between populations. To this end, we examined AF differences in Pop-CMR-associated SNPs between EUR (*n* = 503) and AFR (*n* = 661) populations using data from the 1000 Genomes Project Phase 3 (see the “Investigation of the population specificity of SNPs for Pop-CMRs” section in Materials and methods) [50]. Of the 52 SNPs identified (one SNP was associated with two Pop-CMRs), 36 (69.2%) showed significant AF differences between EUR and AFR populations (|AF difference| > 0.1; Fisher’s exact test, FDR < 0.05) (Table 2). This represents an approximately 2-fold enrichment over the expected number of SNPs if AF differences occurr randomly across the genome, suggesting enrichment of Pop-CMR-associated SNPs at loci with population-specific AFs (69.2% *vs*. 35.0%; 1000 bootstrap replicates, *P* < 0.001) (Figure 3F).

Further, to verify that the genetic influence on the population specificity of DNAm is due to population-specific AFs of the SNPs rather than the SNPs themselves, we categorized Pop-CMRs into two groups based on the AF differences of their associated SNPs between populations: those associated with SNPs showing significant population-specific AFs, and those associated with SNPs showing non-significant AF differences. Each group was then compared to SNP-independent Pop-CMRs. The results revealed that Pop-CMRs associated with SNPs showing significant population-specific AFs exhibited significantly greater between-population DNAm differences than SNP-independent Pop-CMRs (0.41 *vs.* 0.32; Mann–Whitney U test, FDR = 3.4 × 10^−4^), whereas Pop-CMRs associated with SNPs showing non-significant AF differences did not show a significant difference (0.35 *vs*. 0.32; Mann–Whitney U test, FDR = 0.589) (Figure 3G). This suggests that the role of genetic variants in population-specific DNAm may be largely attributable to AF differences.

Notably, population-specific SNP–Pop-CMR associations (*e.g*., thoese present in AFR but absent in EUR) may represent another model by which genetic variants influence population-specific DNAm. However, due to the lack of genotype data for the EUR WGBS samples, we did not examine this model in the present study. Altogether, our findings suggest a substantial role of genetic variation in the population specificity of DNAm.

### SNPs associated with Pop-CMRs were linked to metabolism and infection

Understanding the link between DNAm-associated genetic variants [known as methylation quantitative trait loci (mQTLs)] and human phenotypes can highlight disease-relevant pathways and biological contexts [9,52]. Here, we evaluated the potential impacts of 707 Pop-CMR-associated SNPs on complex traits by overlapping them with genome-wide association study (GWAS) hits obtained from the GWAS Catalog (release date: 30 January 2023) [53]. GWAS hits were filtered using the standard genome-wide significance threshold of *P* < 5.0 × 10^−8^, and all SNPs in high linkage disequilibrium (*r*^2^ ≥ 0.8) with these GWAS hits in AFR populations were extracted using data from the 1000 Genomes Project Phase 3 [50].

We found that 20 SNPs associated with 6 Pop-CMRs overlapped with GWAS hits related to diverse traits, such as obesity and cardioembolic stroke (Table S18). For instance, SNP rs538656 (associated with Pop-CMR chr18:57842408–57842698) showed a notable AF difference between EUR and AFR populations (AF difference = 0.171; Fisher’s exact test, FDR = 5.9 × 10^−10^), and has been previously linked to obesity susceptibility [54]. Based on 1000 bootstrap replicates, these SNPs exhibited stronger enrichment for GWAS traits, such as obesity and cardioembolic stroke, compared to randomly sampled SNPs (Table S19).

Additionally, we conducted functional enrichment analysis on 32 genes whose promoters or gene bodies overlapped with SNPs associated with Pop-CMRs (Table S20). Using an FDR threshold of 0.05, these genes were significantly enriched in 170 GO terms, 1 KEGG pathway, and 2 Reactome pathways, each containing at least 5 tested genes (Table S21). The GO terms were futher classified based on ancestor GO terms [47], with the top 3 being metabolism process, biosynthesis process, and nucleobase-containing compound metabolic process (Figure S7). These findings were consistent with our earlier findings involving genes whose promoters or gene bodies overlap with Pop-CMRs, as well as genes whose expression is associated with Pop-CMR DNAm. Similar enrichment was also observed for infection-related biological processes, such as cellular response to stimulus (GO:0051716) and regulation of response to stimulus (GO:0048583) (Table S21). The overlap in biological processes across comparisons suggests possible links between population-specific DNAm, genetic variation, and complex traits.

### A subset of Pop-CMRs showed population specificity beyond the test populations

**Figure 4:**
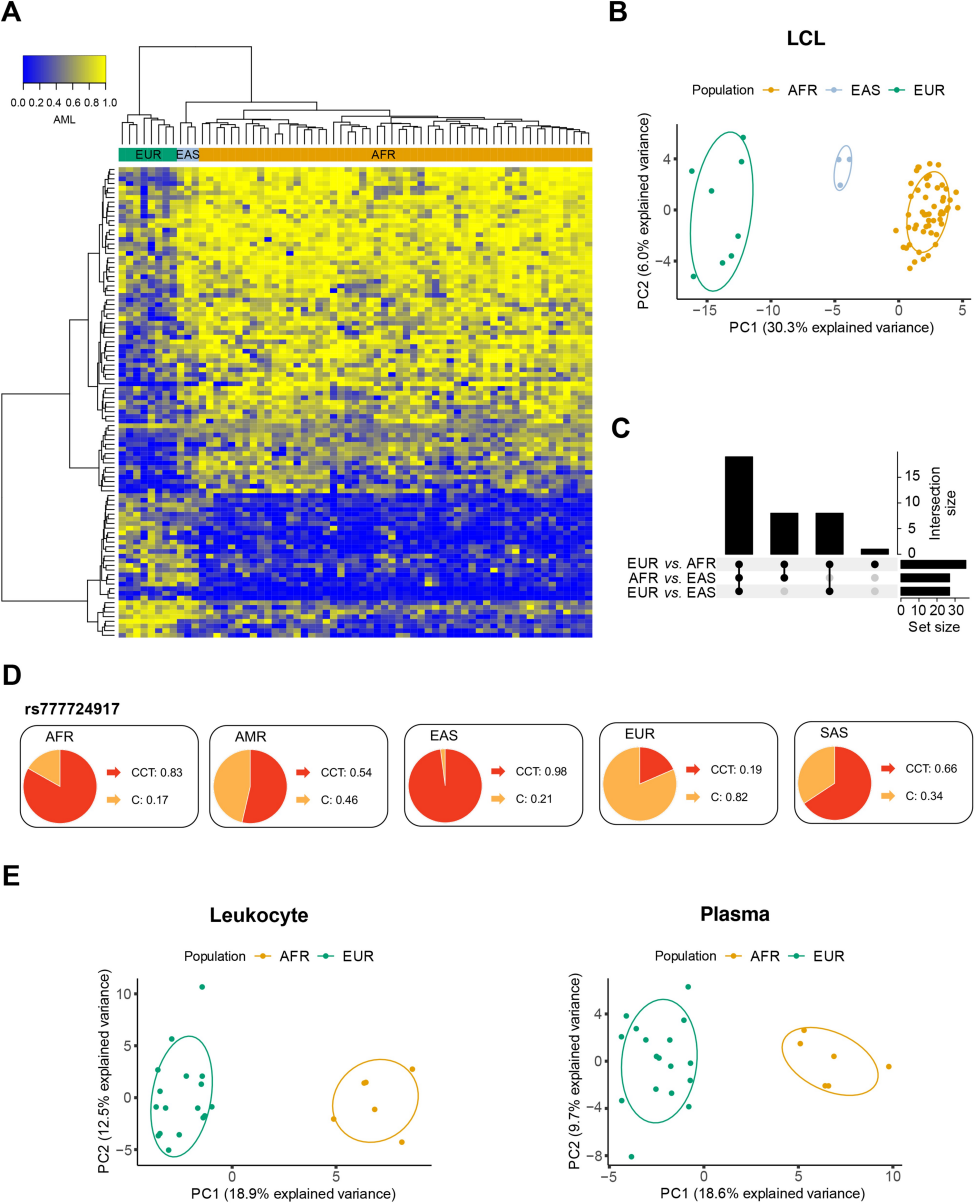
Expansion of 101 Pop-CMRs to the EAS population and blood-based samples **A**. Unsupervised hierarchical clustering heatmap of DNAm patterns at 101 Pop-CMRs across EUR, EAS, and AFR LCL WGBS samples. Columns represent individuals and rows represent Pop-CMRs. Clustering was based on Euclidean distance and complete linkage. **B**. PCA of AMLs at 101 Pop-CMRs across LCL WGBS samples (AFR *n* = 54, EUR *n* = 8, EAS *n* = 3). Each point represents an individual sample, colored by population, with ellipses denoting 95% confidence intervals. **C**. UpSet plot showing the number and overlap of Pop-CMR-associated SNPs with significantly different AFs (|AF difference| > 0.1, FDR < 0.05) between pairs of populations from the 1000 Genomes Project Phase 3 (EUR *n* = 503, AFR *n* = 661, EAS *n* = 504) [50]. **D**. Pie charts showing the AFs of rs777724917 in five human populations. Data are derived from the 1000 Genomes Project Phase 3 [50]. The reference allele is “CCT” and the alternate allele is “C”. **E**. Loadings using the first two PCs of 101 Pop-CMRs for each leukocyte WGBS sample (EUR *n* = 17, AFR *n* = 6) and each plasma WGBS sample (EUR *n* = 17, AFR *n* = 6) on PCA color-coded by ancestry. AMR, Ameriacan; SAS, South Asian.

We explored whether the population specificity of Pop-CMRs could be extended to other human populations in the 1000 Genomes Project Phase 3 [50], such as the East Asian (EAS) population (**Figure 4**A). To this end, three EAS LCL WGBS samples were retrieved from GSE186383 (Table S1) [24]. PCA using the 101 Pop-CMRs revealed that EUR, AFR, and EAS populations formed distinct clusters (Figure 4B). We found that the population clustering was not due to chance, as PCA using 1000 random sets of CMRs showed no population-based clustering. We further examined DNAm differences in Pop-CMRs between EAS and EUR samples, as well as between EAS and AFR samples. Thirteen Pop-CMRs showed significant DNAm differences between EAS and AFR populations (|AML difference| > 0.1; Mann–Whitney U test, FDR < 0.05) (Table 1). Although there were no significant DNAm differences between EAS and EUR populations, likely due to the limited number of samples from both populations, 72, 41, and 15 Pop-CMRs exhibited absolute AML differences greater than 0.1, 0.2, and 0.3, respectively. These results suggest that a subset of Pop-CMRs may show population specificity across all three major human populations (Table 1).

As SNPs with AF differences between EUR and AFR populations play a substantial role in population specificity of Pop-CMRs, we speculated that the 52 SNPs associated with Pop-CMRs may also show AF differences between EAS and EUR/AFR populations and thus present EAS-specific DNAm. Using genotype data from the 1000 Genomes Project Phase 3 (EUR *n* = 503, AFR *n* = 661, EAS *n* = 504) [50], we discovered that 27 of these SNPs showed significant AF differences between EUR and EAS populations, with another 27 showing significant AF differences between AFR and EAS populations (|AF difference| > 0.1; Fisher’s exact test, FDR < 0.05) (Table 2). Of these, 19 SNPs exhibited significant AF differences among the three populations and may underlie the observed DNAm differences between EAS and AFR populations (5 of 13 Pop-CMRs with significant DNAm differences) or between EAS and EUR populations (7 of 15 Pop-CMRs with |AML difference| > 0.3) (Figure 4C). These results provide genetic context for the potential value of Pop-CMRs in distinguishing between these three populations.

We further examined the AF differences of 52 Pop-CMR-associated SNPs in two additional populations from the 1000 Genomes Project Phase 3 [50]: American (AMR *n* = 347) and South Asian (SAS *n* = 489). Using |AF difference| > 0.1 and Fisher’s exact test FDR < 0.05, we observed significant AF differences for 9, 32, and 22 SNPs when comparing AMR with EUR, AFR, and EAS, respectively (Figure S8). Additionally, 12, 33, and 23 SNPs showed AF differences when comparing SAS with EUR, AFR, and EAS, respectively. Notably, SNP rs777724917 (corresponding to Pop-CMR chr1:1329689–1329749) displayed significant AF differences across all five populations (alternative allele C frequencies: 0.815 in EUR, 0.168 in AFR, 0.021 in EAS, 0.464 in AMR, and 0.343 in SAS) (Figure 4D). Given their associations with Pop-CMRs, it will be important to investigate whether Pop-CMRs show specific DNAm patterns in AMR and SAS populations.

### Subsets of Pop-CMRs showed population specificity in peripheral blood leukocyte and plasma cell-free DNA samples

To assess the stability of Pop-CMRs in different tissues, peripheral samples were investigated, as these are arguably more accessible and so are more likely to be used in future studies. The previously identified 101 Pop-CMRs were evaluated in peripheral blood leukocyte and plasma cell-free DNA (cfDNA) samples from 23 healthy adult individuals (GEO: GSE186888; EUR *n* = 17, AFR *n* = 6) who were not included in the initial Pop-CMR discovery dataset (Table S1) [55]. In blood leukocyte samples, PCA revealed that the two populations formed distinct clusters, with PC1 driven by the Pop-CMRs explaining 18.9% of the variance (Figure 4E). This clustering was unlikely due to chance, as PCA using 1000 random sets of CMRs showed no population clustering. Among the 101 Pop-CMRs, 22 displayed significant DNAm differences between the two populations (|AML difference| > 0.1; Mann–Whitney U test, FDR < 0.05) (Table S22).

Similar results were observed in plasma cfDNA samples (Figure 4E). PCA again revealed distinct population clusters, with PC1 accounting for 18.6% of the variance. As with leukocyte data, PCA of 1000 randomly sets of CMRs showed no population clustering. In this context, 21 of the 101 Pop-CMRs exhibited significant DNAm differences between the two populations (|AML difference| > 0.1; Mann–Whitney U test, FDR < 0.05), with 17 of these also identified in leukocyte samples (Table S23). Together, these findings suggest that a subset of Pop-CMRs identified in LCLs may be extended to blood-based samples.

Among the 22 and 21 Pop-CMRs showing significant DNAm differences in leukocytes and plasma, 16 and 15 were, respectively, associated with SNPs in LCL samples, and these SNPs showed AF differences between EUR and AFR populations (Tables S7 and S18).

## Discussion

Comprehensively understanding genetic diversity, including epigenetic variation, is of fundamental importance to both anthropological and medical sciences [2–5]. Initial studies have revealed that epigenetic variation meaningfully contributes to differences between human populations [9–16]. However, current understanding of epigenomic diversity between human populations is limited, as only a small fraction (∼ 3.0%) of genome CpG sites have been investigated to date. To address this limitation, DNAm differences between EUR and AFR populations using WGBS data were investigated. We further leveraged co-methylation patterns across adjacent CpGs to improve statistical power, which was especially critical given the modest sample sizes. This analysis identified 101 Pop-CMRs located in genes with potential functions related to metabolism and infection, where DNAm differences between populations were partly explained by underlying genetic variation. Notably, the Pop-CMRs identified between EUR and AFR LCL samples displayed strong clustering performance for these populations and extended to distinguish individuals from an EAS population as well as from peripheral blood-based tissues (*i.e.*, leukocytes and plasma). These findings will help improve our understanding of epigenomic diversity between human populations, and thus facilitate the development of tools for inferring population structure.

The major advantage of this study was the use of the large number of CpGs measured by WGBS for the discovery of population-specific DNAm patterns. This approach identified 91 Pop-CMRs that have not been evaluated previously in array-based investigations. More importantly, DNAm patterns in four genes (*CCDC42*, *GYPE*, *MAP3K20*, and *OBI1*) were identified exclusively here as being population-specific. These genes have been associated with various diseases that differ in prevalence and incidence across populations, such as type 2 diabetes mellitus [39], susceptibility to malaria infection [41], Alzheimer’s disease [40], and large artery stroke [41]. These findings expand our knowledge of the role of epigenetics in population specificity and provide novel insights into disease susceptibility and outcomes.

These results demonstrated the contribution of genetic variation to population-specific DNAm patterns, as more than half of Pop-CMRs were found to be associated with genetic variants, exhibiting compounded between-population differences in DNAm. This may result from genetic variation or genotype–environment interactions [10,11]. Here, a possible mechanism was suggested in which genetic variation may drive population specificity of DNAm via AF differences between populations, as noted in previous array-based studies and now extended to WGBS-based analyses [10,11,14]. More importantly, this mechanism may provide a genetic context for extending the population specificity of Pop-CMRs to other populations and tissues. As evidenced herein, population specificity of Pop-CMRs was successfully validated in the EAS population and in two peripheral blood-based tissues, with empirical evidence that genetic variants can at least partly underlie these associations. Although the lack of genotype data for the peripheral blood samples prevented the validation of SNP–Pop-CMR associations in these tissues, previous studies have reported relatively low blood cell specificity for mQTLs [56,57]. As such, it is reasonable to speculate that the observed extension of Pop-CMRs to leukocytes and plasma may, at least in part, be due to the cross-tissue influence of population-specific genetic variants on DNAm. Although findings regarding the extension of Pop-CMRs into new populations and tissues were not based on large datasets, the proposed genetic mechanism of population-specific DNAm lends confidence to these conclusions, can inform future studies, and may ultimately support the development of tools for more accurate ancestry prediction based on this method.

While the relationship between Pop-CMRs and metabolic and infectious phenotypes were not examined directly in this study, multiple lines of indirect evidence suggest potential associations. Functional enrichment analysis of genes mapped by Pop-CMRs, regulated by Pop-CMRs, or linked through Pop-CMR-associated SNPs all indicated considerable enrichment in metabolism- and infection-related biological processes or pathways. Furthermore, Pop-CMR-associated SNPs that overlapped with GWAS hits were linked to obesity and cardioembolic stroke, the risk of which can be increased by infectious diseases [58]. Taken together, these observations suggest possible associations between Pop-CMRs and metabolic and infectious phenotypes. Given that immune responses differ between human populations, these associations may reflect population-specific immune responses against infectious pathogens, particularly mediated by B-cells, whose functions are modulated by metabolic processes [2,59].

The WGBS data used in this study came from different laboratories, thereby introducing a risk of technical variation. To address this, several approaches were applied to ensure that any population-specific findings were robust. First, non-Pop-CpGs (see Materials and methods) were used to estimate and adjust for batch effects. Second, global CMR DNAm was determined to be very similar between EUR and AFR populations, indicating that minimal to no systematic technical artifacts, such as batch effects or sample processing differences, affected comparisons between sample populations. Third, Pop-CMRs overlapping with array probes were mostly identified in previous studies, further suggesting the reliability of our approach (Table S8) [9,16,35].

Although we identified CMR DNAm signatures unique to and discriminatory for EUR and AFR populations using the WGBS datasets and validated a subset of these signatures in a DNAm array dataset with 326 samples (EUR *n* = 157, AFR *n* = 169), Pop-CMR identification was still limited by the small sample size in this study. Further efforts to expand the EUR cohort would be essential to validate and reinforce these findings. Furthermore, we assessed epigenetic changes across only three populations, and extrapolation of these results to other populations will necessitate further analyses incorporating greater genetic diversity. Due to the small sample sizes, we were unable to fully represent the individual or subpopulation diversity within each of the three populations studied. It will be necessary to confirm whether these DNAm differences are robust across all individuals of EUR, AFR, and EAS ancestry. In addition, this study investigated and validated DNAm differences only within LCLs and two peripheral blood-based tissues, even though DNAm variation is known to be highly dependent on tissue and cell type [60,61]. We focused on SNPs when exploring the association between genetic variants and DNAm, as it is difficult to accurately detect structural variants (SVs) using short reads, despite individual SVs being more likely to affect gene function than individual SNPs. Further studies utilizing additional DNAm datasets with larger sample sizes, greater population diversity, and broader tissue coverage are required to obtain a more complete picture of pan-tissue DNAm pattern differences across human populations.

In summary, our study illustrates population-specific differences in whole-genome DNAm among individuals from three major human populations and their associations with genetic variation and biological relevance. This study provides crucial insight into epigenomic diversity across human populations and highlights its potential for inferring population structures.

## Materials and methods

### Study population and design

To analyze DNAm at the whole-genome level, we used published WGBS data from 62 LCL samples, including 54 individuals of AFR ancestry (28 males and 26 females) and 8 individuals of EUR ancestry (4 males and 4 females). The AFR samples were derived from two subpopulations, Yoruba in Nigeria (*n* = 8) and Gambian from Western Division – Mandinka (*n* = 46), while the EUR samples were collected from individuals in the USA (*n* = 5) and Sweden (*n* = 3). Data sources are described in Table S1.

As a first step in our multi-layered approach, we identified and characterized CMRs genome-wide using LCL WGBS samples, with validation in 326 HM450K samples and subsequent functional annotation and enrichment analyses (Table S3). We then identified Pop-CMRs between EUR and AFR WGBS samples, and compared them with findings from previous array-based studies. To assess potential functional relevance, we examined the enrichment of Pop-CMRs in biological processes and pathways. We also evaluated the relationship between genetic variation and population-specific DNAm patterns using matched genotype data from 54 AFR WGBS samples. Functional analyses of SNPs associated with Pop-CMRs included overlap with known GWAS hits and GO/pathway enrichment analyses. Finally, we investigated the broader relevance of Pop-CMRs by assessing their patterns in EAS LCL samples (*n* = 3) and an independent blood-based WGBS dataset (EUR *n* = 17 and AFR *n* = 6, each for healthy leukocyte and plasma samples). Data sources are described in Table S1. This multi-layered approach provides insights into whole-genome DNAm variance across populations and its relationship with genetic variants and functional biology.

### WGBS data information and processing

A total of 70 LCL WGBS samples (EUR *n* = 9, AFR *n* = 58, EAS *n* = 3), 23 healthy leukocyte WGBS samples (EUR *n* = 17, AFR *n* = 6), and 23 healthy plasma WGBS samples (EUR *n* = 17, AFR *n* = 6) were obtained from the NCBI BioProject database (BioProject: PRJNA563344 and PRJNA733656) and the NCBI GEO database (GEO: GSE186383, GSE57471, GSE66285, GSE89213, and GSE49627) for analysis (Table S1). LCLs are generated by EBV transformation of primary B-cells and are widely used in immunology and human genetics research [62]. Sequencing protocols and technical details are provided in the original publications [23–28,55] (Table S1).

WGBS data were preprocessed following the Quality Control, Trimming, and Alignment of Bisulfite-Seq Data Protocol [63]. First, quality control (QC) was performed on raw WGBS data using FastQC (v0.11.7; https://www.bioinformatics.babraham.ac.uk/projects/fastqc/). To remove sequencing adapters and low-quality reads, both paired-end and single-end WGBS reads were trimmed using Trim Galore (v0.4.5; https://www.bioinformatics.babraham.ac.uk/projects/trim_galore/). Trimmed reads were then mapped to the human reference genome (GRCh37/hg19) using Bismark (v0.19.0; https://www.bioinformatics.babraham.ac.uk/projects/bismark/) [64] with default parameters. In addition, the deduplication tool in Bismark was used to remove duplications in paired-end and single-end WGBS files. Finally, non-CpG methylation reads were removed using the Bismark filtration tool (parameters: -s for single-end file; -p for paired-end file) before identifying CMRs. Five LCL WGBS samples (EUR *n* = 1, AFR *n* = 4) were excluded due to extremely low genomic coverage or classification as an outlier in PCA.

### Genetic ancestry information confirmation

Genetic ancestry information is avalabe for all LCL samples, except for four individuals enrolled in a previous study from Karolinska Hospital (Stockholm, Sweden) [28]. To confirm the ancestry of these four subjects, we performed random forest prediction based on genotype information extracted from bisulfite sequencing reads by SNP calling in the WGBS data using BS-SNPer, a high-accuracy SNP caller based on a dynamic matrix algorithm and Bayesian statistical framework [20,65]. The following parameters were set to call high-quality SNPs: --minhetfreq 0.1 --minhomfreq 0.85 --minquali 15 --mincover 10 --maxcover 1000 --minread2 2 --errorate 0.02 --mapvalue 10. An average of 6,882,905 SNPs (range: 4,507,103–12,129,165) were detected per paired-end WGBS sample.

A total of 1397 subjects from 11 ethnic groups in HapMap Phase 3 (v3; ftp://ftp.hgsc.bcm.tmc.edu/HapMap3-ENCODE/HapMap3/HapMap3v3) were randomly divided into training and validation datasets at a ratio of 4:1 [66]. Given the genetic similarity among some populations, we combined the CHB (Han Chinese in Beijing, China), CHD (Chinese in Metropolitan Denver, CO, USA), and JPT (Japanese in Tokyo, Japan) into an EAS population, while CEU (Utah residents of Northern and Western European ancestry from the CEPH collection) and TSI (Tuscans in Italy) into a EUR population [66]. Furthermore, three individuals with known ancestry (EUR *n* = 2, AFR *n* = 1) were included in the test dataset as positive controls. Ancestry prediction was conducted using the randomForest R package, implementing Breiman’s random forest algorithm for classification [67]. Based on the prediction accuracy of 0.853 on the validation set, the ancestry of the four individuals without reported ancestry was predicted as EUR, with 100% concordance among positive controls.

### Identification of CMRs from LCL WGBS data

CpGs were extracted from sequence-aligned BAM files within highly mappable regions in the genome using a minimum mapping depth cutoff of 4 in more than half of the samples in each population [68]. Possible batch effects were corrected using 135 CpGs, which were selected based on two criteria: ranked within the top 5% of most stable CpGs between individuals within each population (based on the standard deviation of beta-converted M values [69]), and rankded within the top 5% of most stable CpGs between any two populations (all Mann–Whitney U test, FDR > 0.1) (Table S24). FDR was calculated using the Benjamini–Hochberg method [70]. These CpGs were identified in the DNAm array data of 288 LCL samples (EUR *n* = 96, AFR *n* = 96, EAS *n* = 96) from GSE36369 [12]. Their non-population-specific characteristics were further validated in WGBS samples prior to batch correction (Figure S9). These non-population-specific and inter-individually stable CpGs were then used to estimate and regress out DNAm variation resulting from batch effects among WGBS samples. In addition, 13,462 CpGs previously associated with EBV transformation [28] and 168,914 CpGs located on sex chromosomes were excluded to minimize the influence of EBV transformation and sex chromosome dosage effects on DNAm. After filtering, 14,000,033 CpGs remained for CMR identification.

CMRs were identified as previously described [21]. Methylation correlations between neighboring CpGs within a distance < 400 bp were examined across LCL samples using Pearson correlation (*r*). CpGs with *r* > 0.5 and Benjamini–Hochberg FDR < 0.05 were considered significantly correlated. To reduce the potential effects of the unbalanced sample sizes of the EUR and AFR samples, 1000 bootstrap replicates were performed using all EUR samples and equal-sized randomly sampled AFR subsets. Correlation thresholds were applied as above. To reduce the potential for false positives, only regions containing at least three highly correlated CpG sites were defined as CMRs.

### HM450K datasets for CMR validation

Three HM450K datasets were used to further validate CMRs. The first datatset contained LCL samples retrieved from GSE36369 [12]. This dataset consisted of 96 healthy EUR (Caucasian American) and 96 healthy AFR (African American) subjects from the Human Variation panel (sample sets HD100AA, HD100CAU, HD100CHI; Coriell Cell Repositories), originally collected by the National Institute of General Medical Science (NIGMS) [12]. The second HM450K dataset included 133 HapMap LCL samples derived from GSE39672, containing 60 EUR (Utah residents of Northern and Western European ancestry) and 73 AFR (Yoruba in Ibadan, Nigeria) individuals [14]. The third dataset included five LCL samples downloaded from GSE40699, comprsing four EUR (Utah residents of Northern and Western European ancestry) and one AFR (Yoruba in Ibadan, Nigeria) individuals [30]. Four duplicate samples were removed across all three datasets. The remaining 326 HM450K LCL samples (EUR *n* = 157, AFR *n* = 169) were used for subsequent analysis (Table S3). It should be noted that eight of these samples (EUR *n* = 1, AFR *n* = 7) were subject to WGBS and were included in the LCL WGBS data.

### DNAm array data preprocessing and analysis

For the array datasets used in this study, the downloaded data were normalized using the beta-mixture quantile (BMIQ) normalization method [71]. For each dataset, the 65 SNP control probes, probes on sex chromosomes, probes with missing values in > 5% of samples, and polymorphic CpG probes were removed [37]. After filtering, 413,012 probes shared across the three datasets were retained for analysis. Batch effects among the datasets were corrected using *ComBat* from the sva R package [72]. Pearson correlation analysis was performed to validate the correlation structures of CMRs identified from WGBS data. As in the WGBS analysis, significant correlations between CpGs were defined using *r* > 0.5 and Benjamini–Hochberg FDR < 0.05.

### Detection and validation of Pop-CMRs

WGBS data from 8 EUR and 54 AFR LCL samples were used for genome-wide analysis of CMR DNAm. The DNAm patterns of CMRs were measured as the AML of CpGs within each CMR. Differential AMLs between populations were determined using linear regression with adjustment for sex. FDR was calculated using Benjamini–Hochberg [70]. CMRs with absolute AML difference > 0.1 and FDR < 0.05 were defined as Pop-CMRs [33]. To evaluate the possible effects of unbalanced sample sizes between EUR and AFR samples, 1000 bootstrap replicates were performed on Pop-CMRs using all EUR samples and an equal number of randomly selected AFR samples. As AFR individuals in this study came from two different subpopulations and EUR samples were collected from two different countries, 1000 permutation tests were conducted to examine Pop-CMR DNAm differences between subpopulations. As a result, 35 CMRs that initially passed the AML difference and FDR thresholds were removed due to possible confounding from unequal sample sizes (*n* = 34) or subpopulation structure (*n* = 1). PCA was conducted to visualize Pop-CMR DNAm patterns across samples using the *prcomp* function in the stats R package [73]. Validation of Pop-CMRs in array samples was performed using the Mann–Whitney U test, applying the same AML difference and FDR thresholds mentioned above.

### Enrichment analysis of CMRs, Pop-CMRs, and Pop-CMR-associated SNPs for known genomic elements

To calculate enrichment statistics for CMRs within specific genomic elements, an equal number of genomic regions with matching CpG counts were randomly selected. Random sampling was repeated 1,000,000 times for each genomic element with fold enrichment calculated as the ratio of observed to expected values. Statistical significance was assessed using the empirical *P* values. Enrichment was considered statistically significant at a Benjamini–Hochberg FDR threshold of 0.05. Annotation files for exons, introns, 1-kb downstream regions, 5′-untranslated regions (5′-UTRs), and 3′-UTRs were retrieved from the UCSC Genome Browser (https://genome.ucsc.edu). LncRNA and pseudogene annotations were obtained from the GENCODE database (https://www.gencodegenes.org/releases/19.html) [31]. Predicted regulatory regions based on histone modifications were curated from ENCODE LCL data using the ChromHMM algorithm (http://hgdownload.cse.ucsc.edu/goldenPath/hg19/encodeDCC/wgEncodeBroadHmm) [74]. All genomic coordinates used in this study were based on the human reference genome GRCh37/hg19.

Similarly, 1,000,000 bootstrap replicates were conducted to assess genomic element enrichment among Pop-CMRs and Pop-CMR-associated SNPs. Fold enrichment within each genomic feature was calculated for Pop-CMRs and SNPs by comparing them to randomly selected CMRs and SNPs, respectively. Enrichment was considered statistically significant at a Benjamini–Hochberg FDR threshold of 0.05.

### LCL expression array data preprocessing and analysis

The gene expression array data were retrieved from the Gene Expression Omnibus (GEO: GSE23120, GSE11582, and GSE49628) [28,44,45]. Background correction and normalization were conducted using the robust multi-array average (RMA) method in the oligo R package [75]. For each dataset, QC was performed to remove probes with missing values in > 5% of samples and samples missing > 1% of probes. Batch effects among the three datasets were corrected using the *ComBat* function in the sva R package [72]. After selecting probes shared across datasets, 20,802 probes were retained for further analysis. The association between Pop-CMR methyelation and gene transcription was assessed in a subset of smaples with matched array and WGBS data (*n* = 14) using linear regression. Given this limited sample size, associations with *r* > 0.3 and *P* < 0.05 were considered significant. To emphasize the regulatory potential of Pop-CMRs, we analyzed only those genes whose promoters or gene bodies overlapped with Pop-CMRs [76,77]. Gene expression differences between populations were assessed using the Mann–Whitney U test. Genes with Benjamini–Hochberg FDR < 0.05 were defined as exhibiting population-specific expression.

### Functional enrichment analysis of genes

To investigate the functional significance of the genes of interest, we performed a comprehensive functional enrichment analysis using the g:Profiler toolset (https://biit.cs.ut.ee/gprofiler/gost) [78]. This analysis included three curated databases: GO (molecular functions, biological processes, cellular components), Kyoto Encyclopedia of Genes and Genomes (KEGG), and Reactome. A Benjamini–Hochberg FDR threshold of 0.05 was used to define statistical significance. To illustrate the basic hierarchy and relationships among GO terms in a biological context, the free web tool, CateGOrizer, was used to investigate GO term datasets in terms of the GO classes they represented [47], according to the “GO_Slim2” classification (ftp://ftp.geneontology.org/pub/go/GO_slims/goslim_generic.obo).

### Genotype data preprocessing

WGS genotype data corresponding to 56 WGBS samples (EUR *n* = 2, AFR *n* = 54) were downloaded from the 1000 Genomes Project Phase 3 [50]. QC was conducted using PLINK (v1.90b6) [79], including checks for sex discordance, elevated heterozygosity rate or missing genotype rate, high relatedness, and distinct population stratification. Two EUR individuals with elevated heterozygosity rate or missing genotype rate were removed. SNPs with minor allele frequency (MAF) < 0.01, missing genotype rate > 10%, or Hardy–Weinberg equilibrium exact test *P* < 1.0 × 10^−6^ were excluded [79]. In total, 17,070,886 SNPs from 54 AFR individuals were retained for downstream analysis.

### Mapping of SNP-associated Pop-CMRs

Genetically associated Pop-CMRs were identified by testing SNPs located within a ±10-kb window of each Pop-CMR, consistent with previous studies indicating that *cis*-mQTLs are commonly located near to their associated SNPs [12,51]. The relationship between Pop-CMRs and neighboring SNPs was assessed using a linear additive model with adjustment for sex using the MatrixEQTL R package [80]. Associations were considered significant at a Benjamini–Hochberg FDR threshold of 0.05.

### Investigation of the population specificity of SNPs for Pop-CMRs

To understand how SNPs affect population-specific DNAm levels, the population specificity of 33 SNPs for Pop-CMRs was assessed using unrelated samples (EUR *n* = 503, AFR *n* = 661, EAS *n* = 504, AMR *n* = 347, and SAS *n* = 489) from the 1000 Genomes Project Phase 3 [50]. Population-specific loci were defined as SNPs with significant AF differences between two tested populations based on Fisher’s exact test with |AF difference| > 0.1 and Benjamini–Hochberg FDR < 0.05.

For each random set of SNPs, 33 SNPs were randomly selected from all SNPs (*n* = 17,070,886) that passed QC and were assessed for population specificity using the same approach as described above. Based on 1000 bootstrap resamples, fold enrichment was calculated as the ratio of observed population-specific loci among SNPs associated with Pop-CMRs to those in randomly selected background SNPs. Enrichment was considered statistically significant at an empirical *P* value threshold of 0.05.

### Analysis of the expansion of Pop-CMRs in the EAS population and blood-based samples

Three Han Chinese LCL WGBS samples were collected from GSE186383 [24]. WGBS data were downloaded and preprocessed using the methods described above. Potential batch effects were estimated and regressed out based on non-population-specific and inter-individually stable CpGs. DNAm differences between EAS and EUR, as well as EAS and AFR, were calculated using the Mann–Whitney U test. |AML difference| > 0.1 and FDR < 0.05 were defined as statistically significant [33].

Blood-based samples were collected from GSE186888: leukocytes and plasma [55]. As raw data were not provided, processed BIGWIG files were downloaded. This dataset included peripheral blood leukocyte and plasma samples collected from the same 23 individuals (EUR *n* = 17, AFR *n* = 6). CpGs within each Pop-CMR were extracted, and AML was calculated for each Pop-CMR in each sample. Linear regression with adjustments for sex and age were performed to test Pop-CMR associations with population specificity in leukocytes and plasma, respectively. Associations with |AML difference| > 0.1 and FDR < 0.05 were considered statistically significant.

## Code availability

All scripts used to generate the figures and support the findings of this study are available at https://github.com/functionalepigenomics/Pop-CMR.

## CRediT author statement

**Zheng Dong:** Conceptualization, Data curation, Formal analysis, Investigation, Methodology, Project administration, Resources, Validation, Visualization, Writing – original draft, Writing – review & editing. **Nicole Gladish:** Visualization, Writing – review & editing. **Maggie P.Y. Fu:** Visualization, Writing – review & editing. **Samantha L. Schaffner:** Visualization, Writing – review & editing. **Keegan Korthauer:** Supervision, Visualization, Writing – review & editing. **Michael S. Kobor:** Funding acquisition, Supervision, Writing – review & editing. All authors have read and approved the final manuscript.

## Competing interests

All authors have declared no competing interests.

## Supplementary Material

qzaf096_Supplementary_Data
