# Supplementary material for: Distinct Co-methylation Patterns in African and European Populations and Their Genetic Associations
Source: Genomics Proteomics Bioinformatics. 2025 Mar 22;23(5):qzaf096. doi: 10.1093/gpbjnl/qzaf096 (PMC13005945; doi:10.1093/gpbjnl/qzaf096)
Supplement: qzaf096_Supplementary_Data [file qzaf096_supplementary_data.zip › Supplementary material captions.docx]

## Supplementary material

**Figure S1 Co-methyalted region identification process pipeline**

A total of 62 whole genome bisulfite sequencing (WGBS) samples (European *n* = 8, African *n* = 54) were used for the identification of co-methyalted region (CMRs) after quality control. The alignment and depth cutoff processes were performed separately in each WGBS file. EBV, Epstein-Barr virus.

**Figure S2 Distribution of genes with promoters and gene bodies overlapping CMRs**

**Figure S3 Loadings using the first two principal components of all CMRs for each WGBS LCL sample (African *n* = 54, European *n* = 8) on principal component analysis color-coded by ancestry**

**Figure S4 Enrichment and depletion of 101 population-specific CMRs in genomic elements at nominal significance**

*P* < 0.05. Fold enrichment was calculated based on random sampling repeated 1,000,000 times. For each set of CMRs enriched in a certain genomic element, the same number of CMRs were randomly selected.

**Figure S5 Differential gene expression between European (*n* = 156) and African (*n* = 149)**

**populations.** FDR < 0.05.

**Figure S6 Additional examples illustrating SNP–Pop-CMR correlation**

The correlation between SNP rs6966151 and the Pop-CMR (chr7:105,766,161–105,766,466) is shown on the left, while the correlation between SNP rs543547 and the Pop-CMR (chr6:159,703,533–159,703,779) is shown on the right.

**Figure S7 Pie charts showing Gene Ontology classification for pathway enrichment analysis of 32 genes whose promoters and gene bodies overlapped SNPs associated with Pop-CMRs**

**Figure S8 Upset diagram showing overlap of differential allele frequency of SNPs for Pop-CMRs across three human populations**

A total of 52 SNPs for Pop-CMRs were tested for allele frequency differences between any two of five human populations (EUR *n* = 503, AFR *n* = 661, EAS *n* = 504, AMR *n* = 347, and SAS *n* = 489) in the 1000 Genomes Project. Differential allele frequencies were considered statistically significant between two populations at an allele frequency difference > 0.1 and FDR < 0.05.

**Figure S9 Comparison of distributions of between-population DNA methylation changes among CpGs in WGBS data with and without population specificity**

These non–population-specific CpGs were identified in DNA methyaltion array data (EUR *n* = 96, AFR *n* = 96, EAS *n* = 96) as the top 5% of those stable between individuals across samples in each population and the top 5% of those stable between any two populations.

**Table S1 Characteristics of bisulfite sequencing data sets used in this study**

**Table S2 Characteristics of all CMRs identified from WGBS data**

**Table S3 Characteristics of DNA methylation array data sets used in this study**

**Table S4 Colocalization and enrichment of CMRs in genomic features**

**Table S5 DNAm changes between two human populations across genomic features**

**Table S6 EWAS catalog annotation of CpG sites within Pop-CMRs**

**Table S7 Investigation of Pop-CMRs in an HM450K array data set**

**Table S8 Characteristics of Pop-CMR-Covered array CpGs previously identified as population-specific**

**Table S9 Enrichment of Pop-CMRs in genomic features**

**Table S10 Previously reported population-specific CpGs in the array**

**Table S11 Characteristics of Pop-CMR-Covered array CpGs not previously identified as population-specific**

**Table S12 Two array CpGs overlapping Pop-CMRs were adjacent to previously array-identified population-specific CpGs**

**Table S13 Association between gene expression and Pop-CMR methylation**

**Table S14 Pathway enrichment of genes mapped by Pop-CMRs**

**Table S15 Associations between Pop-CMRs and gene expression in the iMETHYL database**

**Table S16 Pathway enrichment of potential genes modified by Pop-CMRs**

**Table S17 Associations between SNPs and Pop-CMRs**

**Table S18 Association of SNPs for Pop-CMRs with complex traits**

**Table S19 Enrichment of SNPs for Pop-CMRs with complex traits**

**Table S20 Genes whose promoters and gene bodies overlapped the SNPs associated with Pop-CMRs**

**Table S21 Pathway enrichment of genes mapped by SNPs for Pop-CMRs**

**Table S22 Methylation changes of population-specific CMRs (Pop-CMRs) between European and African populations in leukocyte samples**

**Table S23 Methylation changes of population-specific CMRs (Pop-CMRs) between European and African populations in plasma samples**

**Table S24 Non–population-specific and interindividual stable CpGs identified in GSE36369**
