# Supplementary figures and images for: Distinct Co-methylation Patterns in African and European Populations and Their Genetic Associations
Source: Genomics Proteomics Bioinformatics. 2025 Mar 22;23(5):qzaf096. doi: 10.1093/gpbjnl/qzaf096 (PMC13005945; doi:10.1093/gpbjnl/qzaf096)

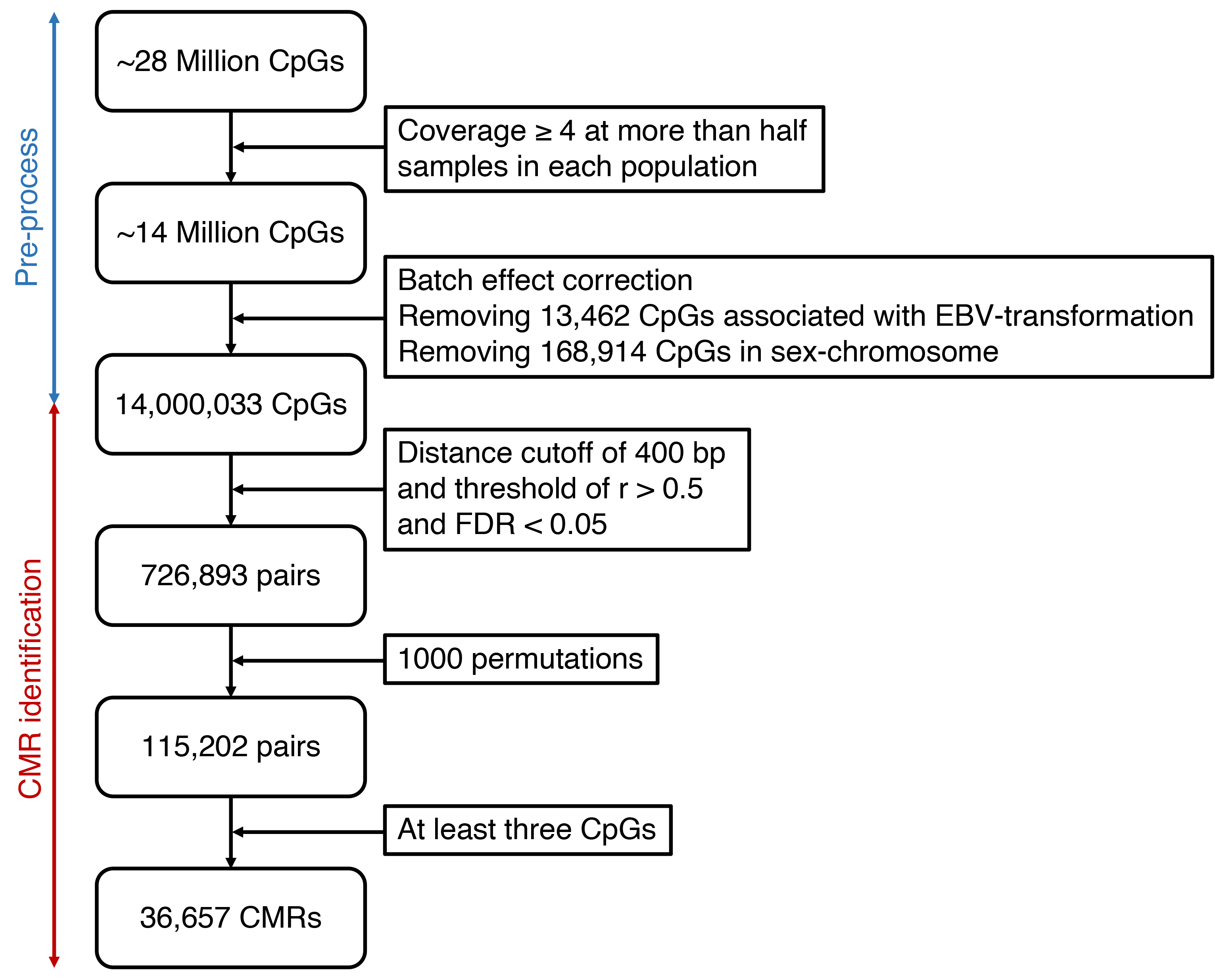

Supplement: qzaf096_Supplementary_Data [file qzaf096_supplementary_data.zip › Figure S1.docx]

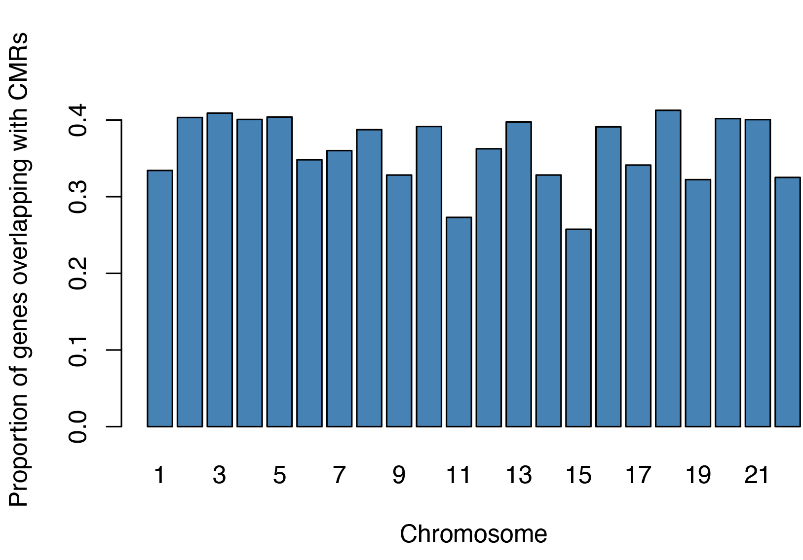

Supplement: qzaf096_Supplementary_Data [file qzaf096_supplementary_data.zip › Figure S2.docx]

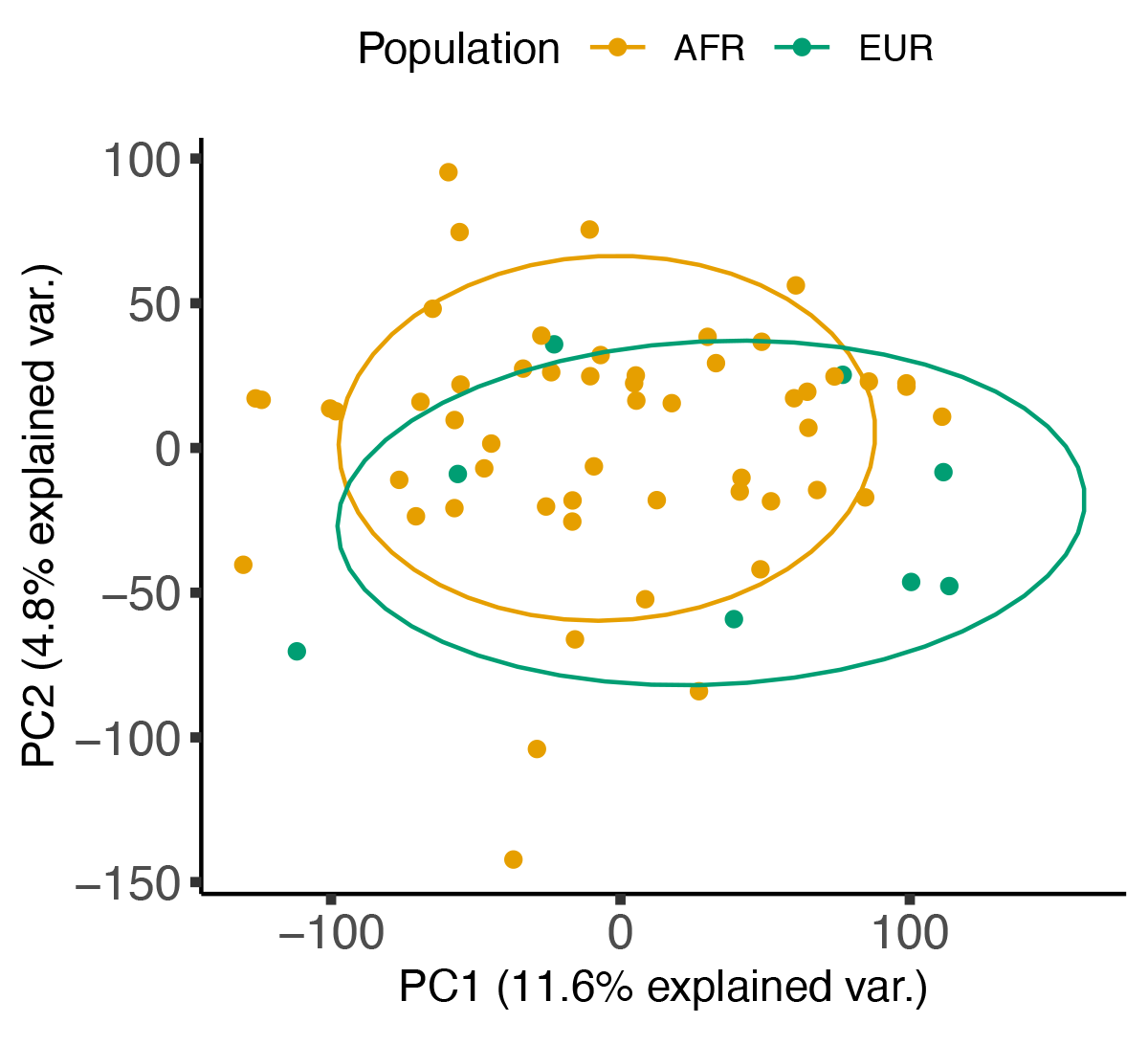

Supplement: qzaf096_Supplementary_Data [file qzaf096_supplementary_data.zip › Figure S3.docx]

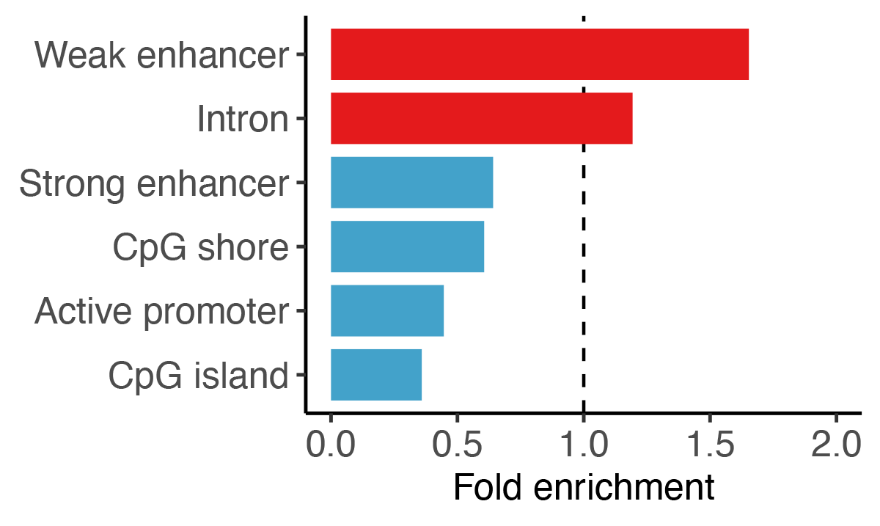

Supplement: qzaf096_Supplementary_Data [file qzaf096_supplementary_data.zip › Figure S4.docx]

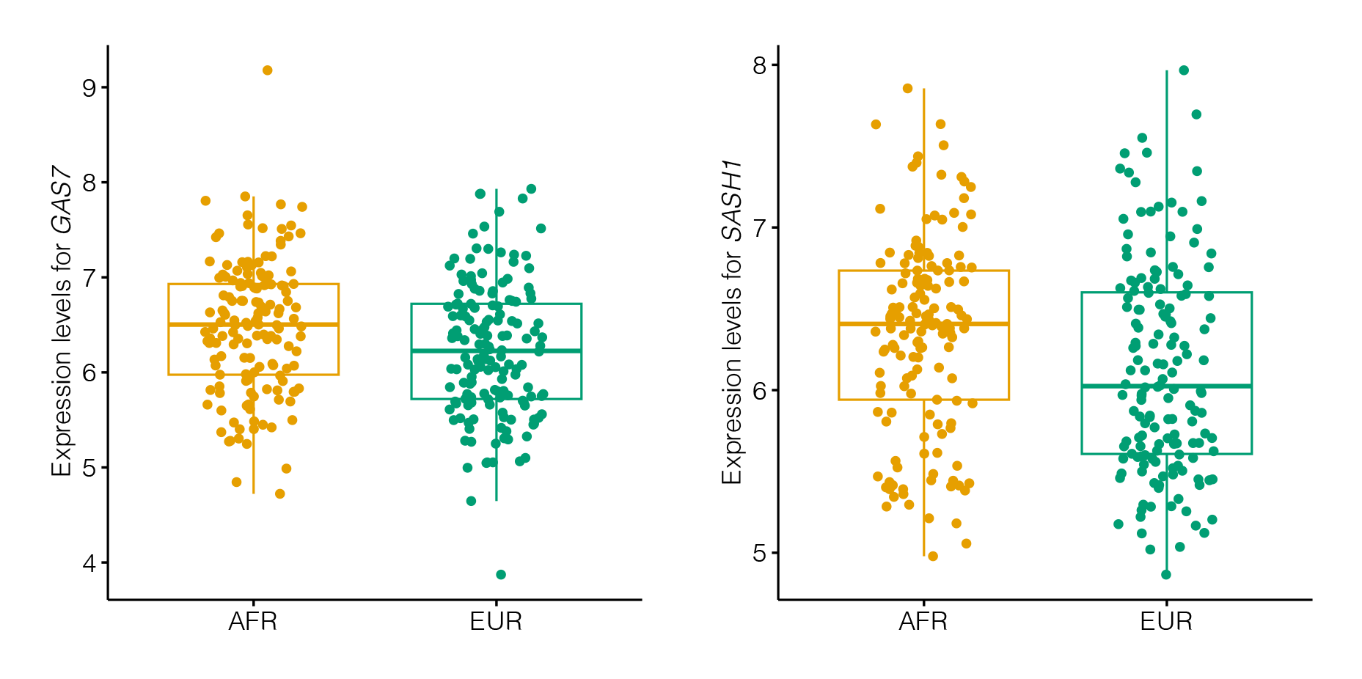

Supplement: qzaf096_Supplementary_Data [file qzaf096_supplementary_data.zip › Figure S5.docx]

**
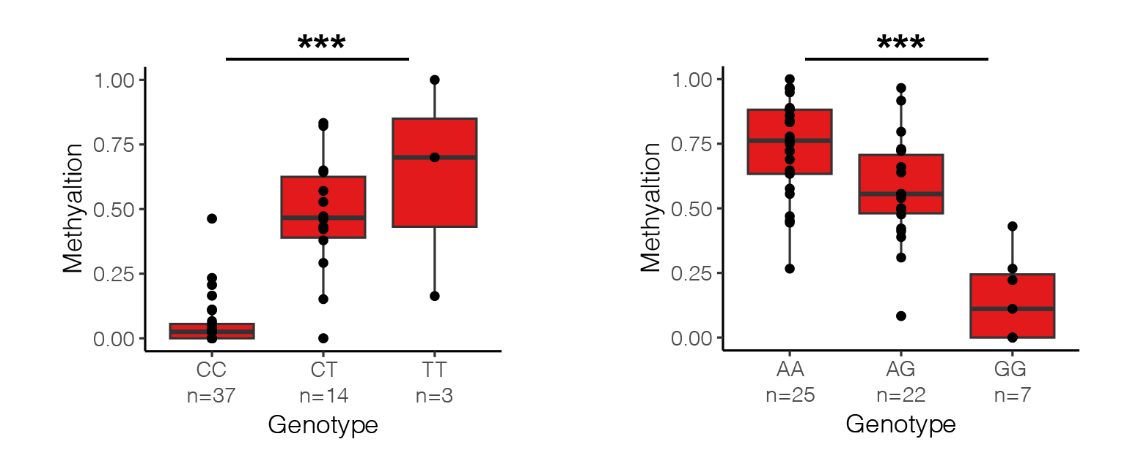
**

Supplement: qzaf096_Supplementary_Data [file qzaf096_supplementary_data.zip › Figure S6.docx]

**
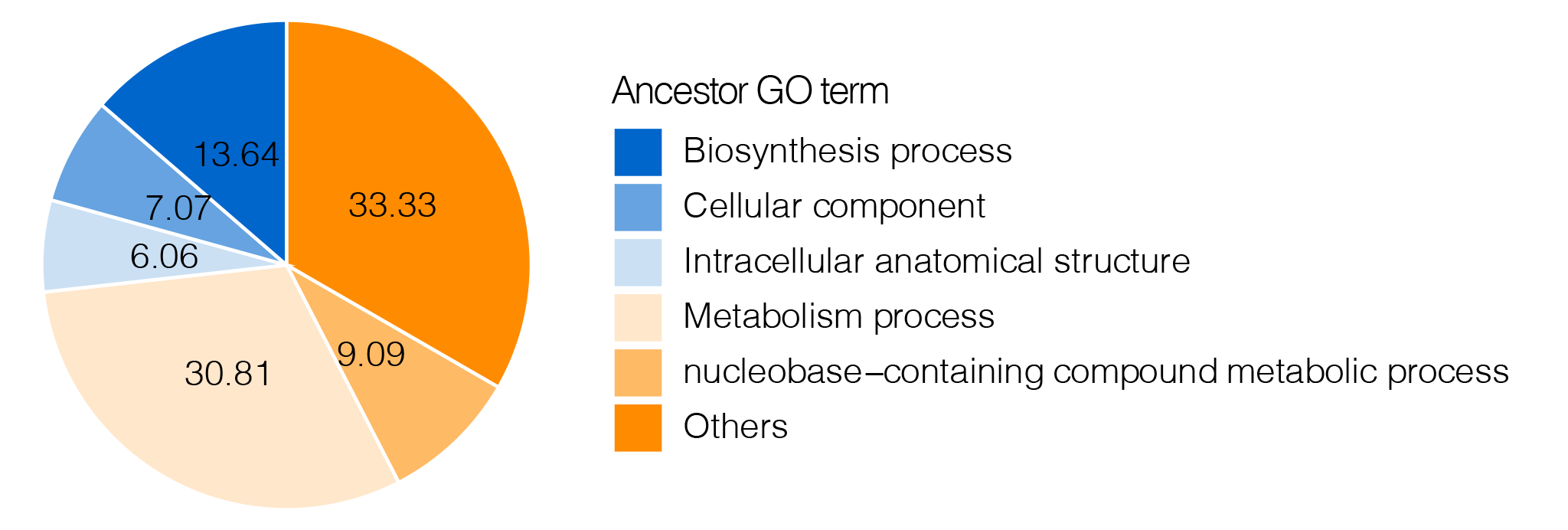
**

Supplement: qzaf096_Supplementary_Data [file qzaf096_supplementary_data.zip › Figure S7.docx]

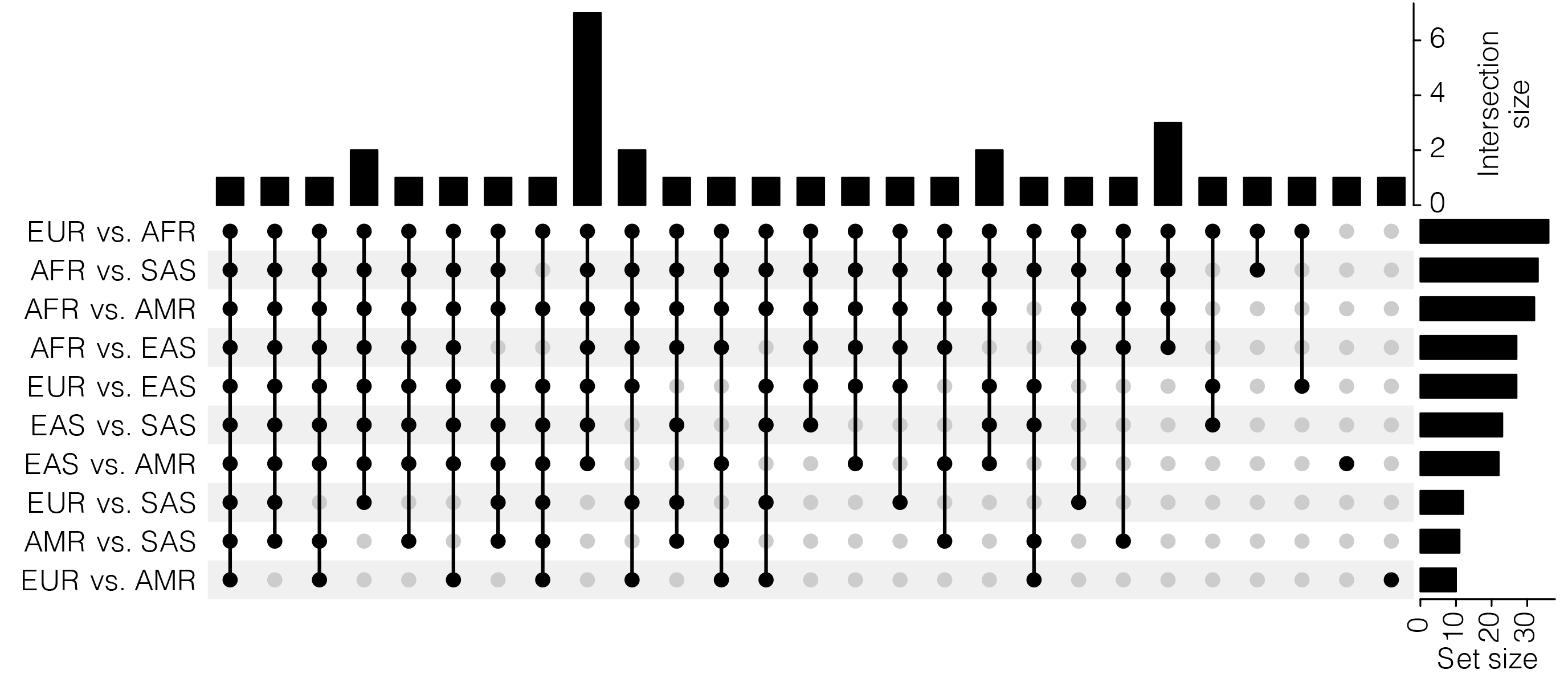

Supplement: qzaf096_Supplementary_Data [file qzaf096_supplementary_data.zip › Figure S8.docx]

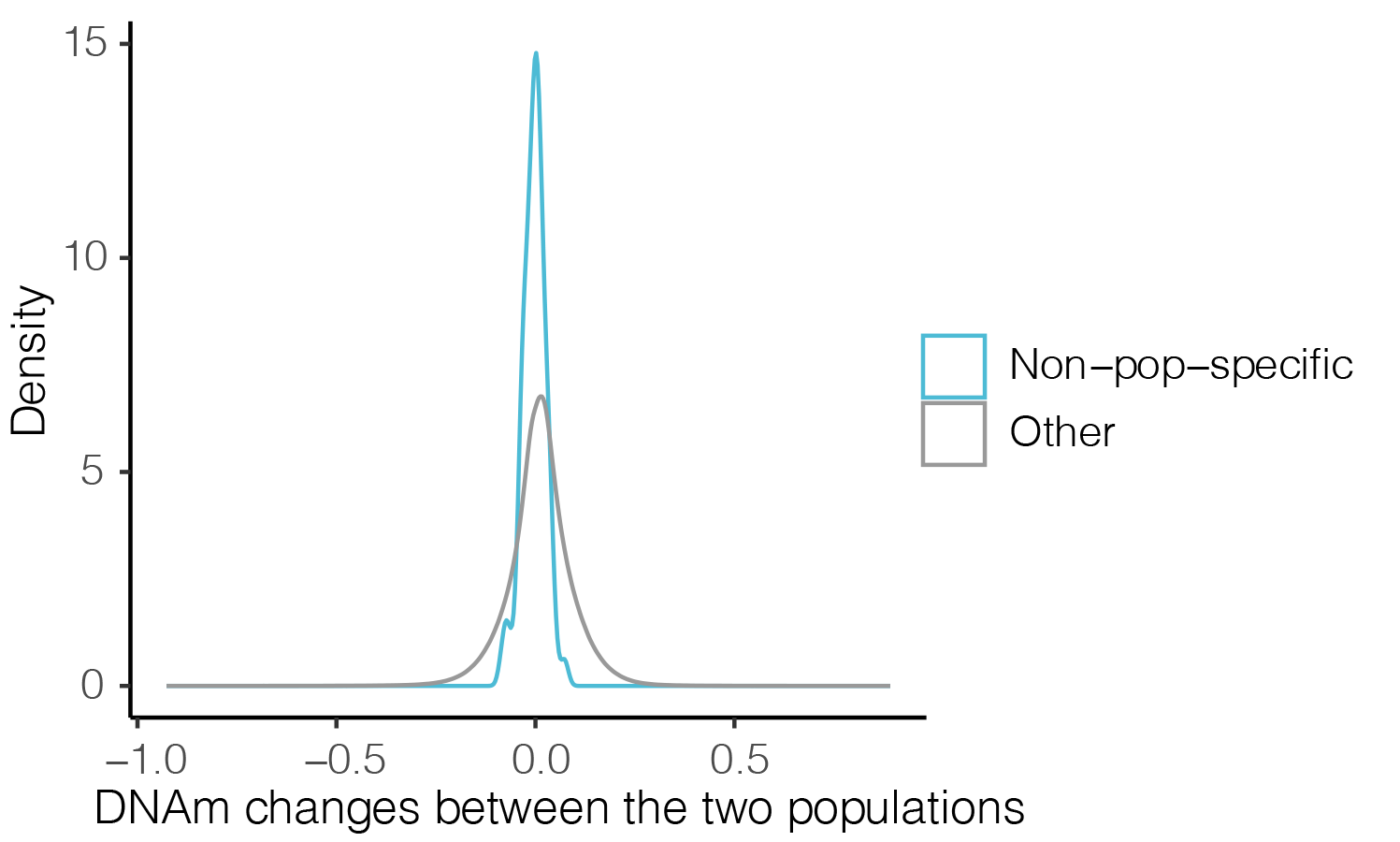

Supplement: qzaf096_Supplementary_Data [file qzaf096_supplementary_data.zip › Figure S9.docx]
